# Unveiling the efficacy and safety of Erenumab, a monoclonal antibody targeting calcitonin gene-related peptide (CGRP) receptor, in patients with chronic and episodic migraine: a GRADE-assessed systematic review and meta-analysis of randomized clinical trials with subgroup analysis

**DOI:** 10.1186/s13005-025-00494-w

**Published:** 2025-03-26

**Authors:** Mohamed E. Haseeb, Hazem E. Mohammed, Hatem Yaser, George Hanen, Mohamed Nasser, Shehab Yaser, Zeyad Bady

**Affiliations:** 1 Faculty of Medicine, Minia University, Minia, Egypt; 2 Faculty of Medicine, Assiut University, Assiut, Egypt; 3 Medical Research Group of Egypt (MRGE), Negida Academy, Cairo, Egypt

**Keywords:** Erenumab, CGRP, Migraine, Efficacy, Safety, Systematic review, Meta-analysis

## Abstract

**Background:**

Migraine is a highly prevalent and disabling disease, affecting nearly 14% of the global population. Preventive medications involve drugs like beta-adrenergic blockers, antidepressants, and anticonvulsants. However, these drugs lacked effectiveness, and patients showed poor tolerance and low adherence to them. Erenumab, a calcitonin gene-related peptide receptor blocker, has recently shown promising results in migraine management. In this meta-analysis, the efficacy of Erenumab is investigated by employing a subgroup analysis approach.

**Methods:**

We conducted a systematic search of six electronic databases until July 2024. Review Manager 5.4 software was utilized for the analysis, based on either weighted mean difference (MD) and standard deviation (SD) for continuous outcomes or risk ratio (RR) for dichotomous outcomes, with a confidence interval (CI) of 95%. A *P*-value < 0.05 indicated statistical significance. The study was registered on PROSPERO with registration number CRD42024573300. Additionally, we conducted subgroup analyses and assessed the quality of evidence using GRADE.

**Results:**

A total of 20 randomized controlled trials (*n* = 5212) were included in our analysis. At three months, Erenumab showed statistically significant improvements in monthly migraine days (MMD), monthly acute migraine-specific medication days (MSMD), Headache Impact Test (HIT-6) score, and ≥ 50% reduction from baseline in MMD (MD: -1.78, 95% CI: [-2.37 to -1.20], *P* < 0.00001), (MD: -1.36, 95% CI: [-1.92 to -0.81], *P* < 0.00001), (MD: -2.83, 95% CI: [-3.83 to -1.82], *P* < 0.00001), and (RR: 1.52, 95% CI: [1.31 to 1.76], *P* < 0.00001), respectively. Subgroup analysis revealed that Erenumab was significantly more effective in patients with prior preventive treatment failures compared to patients with no prior failure. No significant difference in Erenumab`s response existed between episodic and chronic migraine or between 140 and 70 mg, except for MSMD in dose subgrouping. Only constipation emerged as a significant adverse effect in the Erenumab group.

**Conclusions:**

This meta-analysis found that Erenumab significantly reduced migraine attack frequency, medication days, and physical impairment. It was more effective for patients with prior treatment failures. The 140 mg dose showed better MSMD reduction than 70 mg. Erenumab's safety profile was similar to that of placebo, with only constipation noted as significant.

**Supplementary Information:**

The online version contains supplementary material available at 10.1186/s13005-025-00494-w.

## Background

Migraine is a neurological disorder characterized by headaches of moderate to severe intensity, possibly accompanied by an aura. The pain is usually unilateral, pulsatile, and associated with sensitivity to light and sound [1]. Migraine is a highly prevalent and disabling disease. It affects approximately 14% of the global population and caused over 45.1 million years of life lived with disability in 2016 [2, 3]. Despite the high prevalence and burden, the disease pathophysiology remains poorly understood. Besides, the pharmacotherapy of the disease encounters many challenges, with few drugs showing evidence-based efficacy in management and prevention [4–6]. Preventive strategies often involve the use of drugs such as beta-adrenergic blockers (like propranolol), certain antidepressants (such as amitriptyline), and anticonvulsants (like topiramate) [7]. These drugs, however, were not originally developed to treat migraines, and their exact mechanisms of action in preventing migraine attacks remain unclear [7, 8]. As many as 50% of patients report inadequate effectiveness or poor tolerance of these treatments, leading to early cessation of therapy [9–12]. Accordingly, a significant number of individuals struggle to control their migraines with current preventive options, leading to high levels of disability and a significant reduced quality of life [13]. Furthermore, low adherence rates (81% of patients had gaps of > 90 days in their migraine prevention in the first year) and low persistence (20% of patients at 12 months) for oral migraine preventive therapies lead to frequent switching, re-initiation, or complete cessation of preventive therapies across the migraine spectrum. As patients switch between preventive therapies, these discontinuation rates increase [14, 15]. Therefore, novel therapies have recently been proposed for the management of migraine.

Erenumab, a calcitonin gene-related peptide (CGRP) receptor blocker, has recently shown promising results in managing migraine [16–36]. CGRP plays an integral role in the complex pathophysiology of migraine. During a migraine attack, CGRP is released from the nerve terminals of the trigeminal nerve, subsequently causing vasodilation and neurogenic inflammation [37, 38]. The role of CGRP in migraine has been even more prominent when administration of CGRP elicited migraine-like symptoms in susceptible individuals [38, 39].

The present study aims to provide class-one evidence regarding the effectiveness and safety of Erenumab in treating migraines, supported by what we believe is the most extensive meta-analysis to date. Unlike the latest meta-analysis that included only observational studies [40], the pooled results of this study are derived entirely from randomized-controlled trials, providing a more precise insight into the effect of Erenumab in migraine. Besides, our meta-analysis sub-grouped the pooled effect with respect to Erenumab doses, which were not taken into account in other meta-analyses like Fernandez-Bravo-Rodrigo et al. [40]. Moreover, our analysis delved into details not covered in previous relevant studies [41]; our meta-analysis is the first to include a subgroup analysis based on the history of prior failures with migraine-preventive treatments. Additionally, we conducted two further subgroup analyses: one comparing episodic and chronic migraines and another comparing different doses of Erenumab (70 mg versus 140 mg). We addressed several metrics assessing migraine management efficacy, including MMD (Monthly Migraine Days), MSMD (Monthly Severe Migraine Days), and HIT-6 (Headache Impact Test). Furthermore, we aimed to provide insights into the safety profile of the drug. Additionally, we investigated sources of heterogeneity whenever possible using sensitivity analysis and assessed the quality of evidence using GRADE (Grading of Recommendations, Assessment, Development, and Evaluations).

## Methods

This systematic review and meta-analysis followed the criteria of the Preferred Reporting Items for Systematic Review and Meta-analysis (PRISMA) statement [42]. The protocol held a registration number CRD42024573300 on PROSPERO.

### Eligibility criteria

The included studies followed the following criteria:

1. randomized controlled trials (RCTs);
2. studies including patients diagnosed with migraine: either chronic migraine defined as ≥ 15 headache days/month plus ≥ 8 migraine days/month for at least three months or episodic migraine defined as 4 to < 15 migraine days per month and < 15 headache days per month for at least three months before screening and during the baseline period of the trial [1];
3. the intervention group in the participating RCTs was Erenumab 70 mg or 140 mg, and the comparator was placebo with a minimum follow-up period of 3 months;
4. English-language studies only.

Observational studies, case reports, conference abstracts, uncontrolled studies, and studies not written in English were excluded.

### Search strategy

Comprehensive research was conducted from inception until July 2024 in Scopus, PubMed, WOS, Embase, Clinical trials.gov, and Cochrane Central Register of Controlled Trials (CENTRAL) databases. The search strategy comprised specific keywords and Medical Science Heading (MeSH) terms, including the following: "Erenumab," "Erenumab-aooe," "AMG334," "headache*," "migraine*," and "Cephalgia."

### Study selection data extraction

After developing the search strategy, two authors independently performed studies screening using Rayyan online software [43]. We began initially with title-abstract screening, followed by full-text screening. A third reviewer was responsible for resolving conflicts between the two authors in the inclusion process. Four authors have extracted data independently on an online Excel sheet for easier access and communication between authors. The online sheet included study characteristics, population baseline characteristics, and outcome measures data. Study characteristics included study name and year, sample size, design, duration of treatment, population, and the key findings. Population baseline characteristics included sample size, age, gender, history of use or failure of prior migraine-preventive treatment, and duration of migraine. Outcome measures involved:

1. MMD (Monthly Migraine Days) refers to the number of days per month a person experiences migraine headaches and either at least two pain characteristics (unilateral, throbbing, moderate to severe, or aggravated by physical activity) and one non-pain symptom (nausea, vomiting, or both photophobia and phonophobia).
2. MSMD (monthly acute migraine-specific medication treatment days) refers to the number of days in a month that a person requires migraine medication (only migraine-specific medications like triptans or/and ergots) [34].
3. HIT-6 (Headache Impact Test) assesses the headaches' impact on quality of life. It comprises six questions that evaluate headache-related disability, covering pain severity, daily activity interference, fatigue, cognitive issues, and emotional distress. Scores vary from 36 to 78, with higher scores reflecting a higher impact [44].
4. MPFID-PI (Migraine Physical Function Impact Diary—Physical Impairment) and MPID-EA (Migraine Physical Function Impact Diary—Everyday Activities) are part of the MPFID, a validated patient-reported outcome tool developed to measure the impact of migraine on physical functioning and daily activities [45].
5. mMIDAS (modified Migraine Disability Assessment) is a simplified questionnaire that assesses the impact of migraine on the quality of life [46].

Our primary outcomes involved MMD, MSMD, HIT-6, and ≥ 50 reduction from baseline in MMD, and our secondary outcomes comprised MPFID-PI, mMIDAS, and the safety of Erenumab.

### Quality assessment

The included (RCTs) quality was assessed using the Revised Cochrane risk-of-bias tool for randomized trials (ROB 2). Five domains were evaluated during the quality assessment: randomization process bias, bias due to deviations from intended intervention, missing outcome data bias, bias in the measurement of the outcome, and bias in the selection of the reported result.

Two reviewers independently assessed each study, and disagreements were resolved by discussion or consultation with a third reviewer. Studies were categorized as having a low risk of bias, some concerns, or a high risk of bias based on the ROB 2 criteria [47].

### Statistical analysis

Review Manager 5.4 software (RevMan) was used for the statistical analysis of both continuous and dichotomous outcomes [48]. We applied the random effect model employing the DerSimonian-Laird method [49]. The statistical analysis was based on a mean difference (MD) and standard deviation (SD) whenever the outcomes were continuous and on risk ratio (RR) when they were dichotomous, with a confidence interval (CI) of 95% and a statistically significant *P*-value was considered if it was < 0.05. The heterogeneity of the included studies was evaluated using the Higgins score (*I*2). I-square values ≥ 50% were indicative of high heterogeneity [50]. Adverse events were reported as the number per study arm and pooled as RR. Furthermore, we conducted sensitivity analyses to assess the heterogeneity and robustness of the results whenever possible. One study adopted a crossover design [35]; therefore, we used the paired analysis method mentioned in the Cochrane Handbook [51].

#### Quality of evidence

The level of certainty of the generated evidence was evaluated using the Grading of Recommendations, Assessment, Development and Evaluation criteria (GRADE) [52, 53] by the GRADEpro Guideline Development Tool (GDT) online tool [54]. GRADE tool assesses the evidence and classifies it into four levels of certainty: very low, low, moderate, and high, taking into consideration the following domains of evaluation: risk of bias, inconsistency, indirect evidence, imprecision, publication bias, and other domains like dose–response effect and plausible confounding.

#### Publication bias

The Luis Furuya-Kanamori asymmetry index (LFK index) and the Doi plot were used to evaluate the publication bias. Publication bias is reflected by the existence of asymmetry, whereas its absence is reflected by symmetry. LFK index values of ≤  ± 1, >  ± 1 but ≤  ± 2, and >  ± 2 indicate no asymmetry, minor asymmetry, and major asymmetry, respectively [55]. Using the MetaXL Version 5.3 [56] add-in for Microsoft Excel, we conducted a publication bias analysis and generated Doi plots for our primary outcomes. Furthermore, the funnel plots using Stata 17.0 software were generated for the primary outcomes.

## Results

### Literature search

**Fig. 1:**
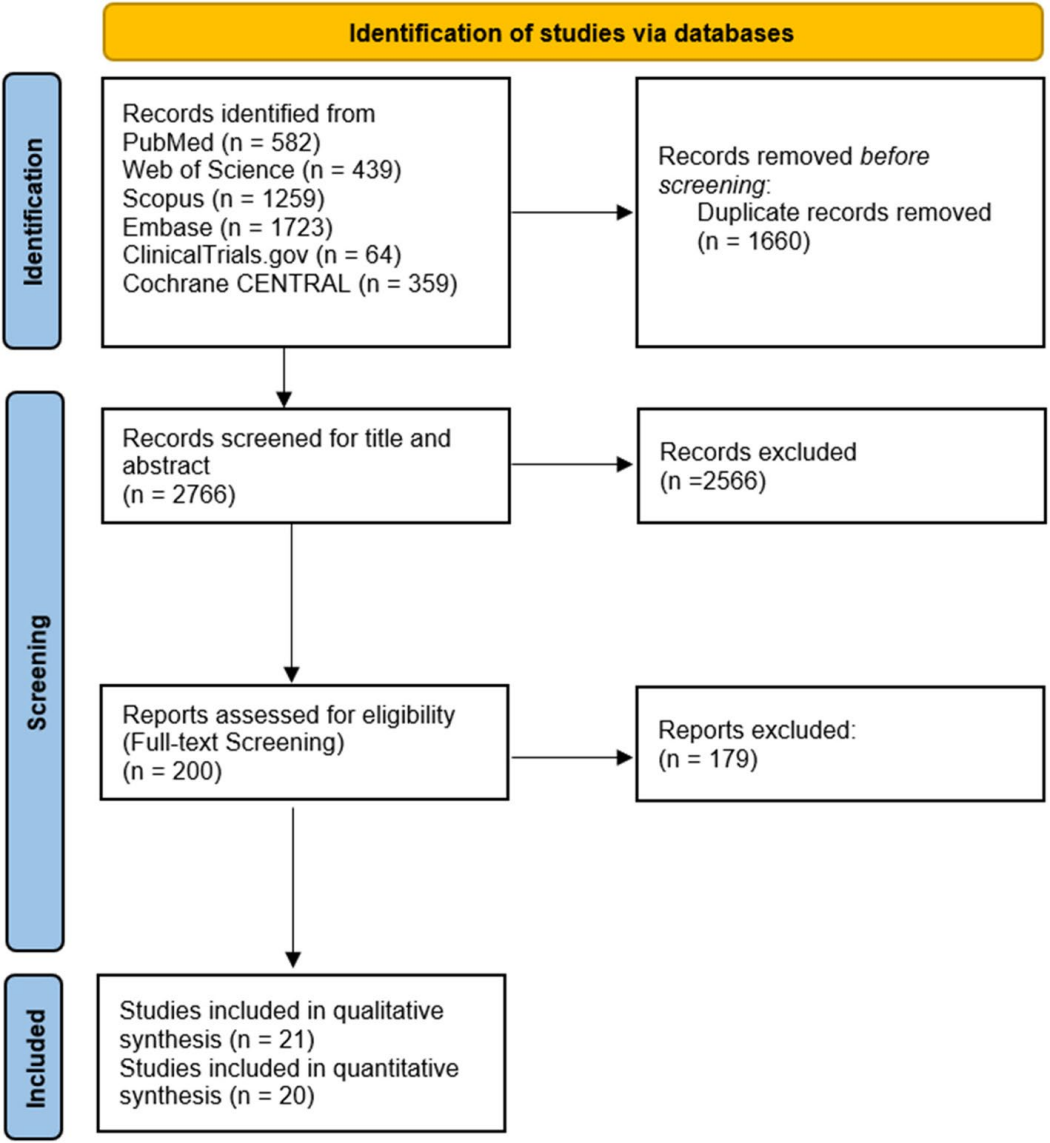
PRISMA flowchart of study selection

We identified a total of 4426 records after conducting our search strategy. Using Endnote, we identified 1660 duplicates and removed them. The remaining 2766 records underwent vigorous title/abstract screening, yielding 200 records left for the full-text screening process. Eventually, 21 RCTs were eligible for our qualitative analysis, and 20 (*n* = 5212) underwent quantitative analysis. The PRISMA flow diagram is shown in Fig. 1.

### Study and population characteristics

A total of 21 [16–36] studies were enrolled in our systematic review and meta-analysis. All the included studies were RCTs, with a total number of participants of 5212. Eight studies were either post-hoc analyses or subgroup analyses based on previously conducted RCTs. The STRIVE study [36] provided the largest number of participants among all studies (*n* = 955), while Hoon et al. [20] provided the smallest number with 12 patients. Most RCTs included three arms comprising placebo and two doses of Erenumab: 140 mg and 70 mg. The majority of the studies (*n* = 16) included patients diagnosed with episodic migraine. Yu et al. (DRAGON) [29] and Tepper et al. [26] studies involved exclusively patients diagnosed with chronic migraine. In addition, Basedau et al. [17], Takeshiama et al. [25], and Hirata et al. [19] studies included patients with either chronic or episodic migraine (mixed). The patients included in most studies exhibited a mixture of no prior treatment failures and previous failures of other preventive treatments. The studies that assessed only patients with previous treatment failures are Filippi et. [35], Reuter et al. (LIBERTY) [22], Hirata et al. [19], Ashina et al. [16], and Goadsby et al. 2019 [18]. The latter three studies provided further details on patient subgroups according to the occurrence of prior treatment failure. The mean age across studies ranged from 24 to 48 years old, with females being the predominant participants in all studies. All study characteristics, including sample size, duration of treatment, and the key findings, are represented in Table 1. Furthermore, the characteristics of the studies’ population are summarized in Table 2.

**Table 1:** Summary of the included study characteristics

Study name	Sample size	Study design	Study setting and duration of treatment	Population	Key findings
**Basedau et al. 2024** [17]	Total: 40 Erenumab 70 mg: 21 Placebo: 19	Double-blind, randomized, placebo-controlled trial	Headache outpatient clinic of the University Medical Center Hamburg Treatment duration: 4 weeks	Adult patients who have been diagnosed with migraine based on the ICHD-3 criteria, are scheduled to receive Erenumab 70 mg as per national guidelines and have not previously used CGRP-antibody treatments	There was a higher decrease in headache frequency in the Erenumab group than the placebo group. Additionally, for the ≥ 50% responder rate, the Erenumab group had better response results
**Filippi et al. 2023** [35]	Total: 61 Erenumab 140 mg: 30 Placebo: 31	Phase 4, randomized, double-blind, placebo-controlled, multicenter trial with a crossover design	This trial took place in five Headache Centers in Italy starting from 30 July 2019 until 5 July 2021. Treatment duration: 24 weeks (patients received either Erenumab 140 mg or placebo for 12 weeks, followed by a 12-week crossover)	Eligible participants are adults aged 18 to 65 who have experienced migraines with or without aura for at least 12 months, with a frequency between 4 to < 15 migraine days per month and headache days fewer than 15 per month over the past three months, as confirmed by a headache diary. Additionally, candidates must have previously failed at least two treatment categories for migraine prevention because of the lack of effectiveness or poor tolerability	Erenumab resulted in a greater reduction in HIT-6 and MMD at month 3 than placebo, with *P* values 0·0001 and < 0·0001, respectively. The only notable treatment-emergent adverse events were constipation and upper respiratory tract infection
**Yu et al. 2022 (The DRAGON Study)** [29]	Total: 557 patients Erenumab 70 mg: 279 Placebo: 278	Phase 3, randomised, double-blind, placebo-controlled study of Erenumab	The trial was conducted at 64 sites in 9 countries in Asia or areas including India, China, Malaysia, the Republic of Korea, the Philippines, Singapore, Thailand, Taiwan, and Vietnam. Treatment duration: 12 weeks	Adults aged between 18 and 65 years with a history of CM, lasting ≥ 12 months before screening, as defined by ICHD-3. To qualify for randomization, patients needed to have a history of ≥ 15 headache days per month, of which at least 8 days have to meet the criteria for migraine days, as recorded in their eDiary during the baseline period	Erenumab exhibited a better reduction in MMD (3 months) than placebo (*P* = 0.015). Additionally, a greater percentage of Erenumab group experienced a ≥ 50% reduction in MMD (3 months) compared to the placebo group (*P* = 0.014). Constipation and upper respiratory tract infections were the most common adverse events
**Chowdhury et al. 2022** [32]	Total: 351 patients Erenumab 70 mg: 133 Erenumab 140 mg: 94 Placebo:124	Post-hoc for The EMPOwER study [27]	The study was conducted across 27 research locations in India Treatment duration: Three months	Adults aged between 18 and 65 with a documented migraine history of at least 12 months and experiencing migraine symptoms on 4 to less than 15 days per month were eligible for inclusion	At month 3, Erenumab 70 mg and 140 mg exhibited a higher reduction in MMD than placebo, with P values equal to 0.174 and 0.164, respectively. The most reported adverse events were pyrexia and nasopharyngitis
**Wang et al. 2021 (The EMPOwER Study)** [27]	Total: 900 patients Erenumab 70 mg: 338 Erenumab 140 mg: 224 Placebo: 338	Phase 3, multicentre, randomised, double-blind, placebo-controlled, parallel-group study	The study was performed from February 8, 2018, to January 13, 2020, at 83 locations in 11 countries in Asia, the Middle East, and Latin America. Treatment duration: a 12-week safety follow-up period comes after a three-month double-blind treatment phase	Adults aged 18–65 with diagnosed migraines as per ICHD-3 beta were eligible. For the three months before screening and during the baseline period, participants had to experience four to fewer than fifteen migraine days per month and fewer than fifteen total headache days per month	At month 3, Erenumab at doses of 140 mg and 70 mg resulted in a better reduction in MMD than placebo, with P values equal to < 0.001 and 0.002, respectively. The most commonly reported adverse events were constipation and pyrexia
**Takishema et al. 2021** [25]	Total: 261 patients Erenumab 70 mg: 130 Placebo: 131	Phase 3, randomized, double-blind, placebo-controlled study in Japanese patients	41 centers across Japan Treatment duration: 24 weeks	Japanese patients aged 20 to 65 who provided informed consent before the study began were eligible to participate. They needed to have a history of migraine for at least 12 months before screening, based on the definition of ICHD-3, supported by medical records and/or patient self-report. Additionally, they must have had either CM or EM during the 3 months prior to the screening	Erenumab 70 mg was effective in reducing MMD compared to placebo over 4 to 6 months for CM patients (*P* = 0.089) and EM patients (*P* < 0.001). It was also effective in achieving ≥ 50% reduction in MMD (*P* = 0.005) and MSMD reduction (*P* < 0.001)
**Lanteri-Minet et al. 2021** [21]	Total: 246 patients Erenumab 140 mg: 121 Placebo: 125	Post-hoc for LIBERTY [22] which was a 12-week, randomised, double-blind, placebo-controlled, phase 3b study	59 sites in 16 countries across Europe and Australia Treatment duration:12 weeks	Adults aged 18 to 65 and have a history of episodic migraine, lasting at least 12 months. They need to have between 4 and 14 migraine days per month over the 3 months before screening while experiencing headaches no more than 14 days per month overall. Additionally, they should have previously failed 2 to 4 preventive migraine treatments	Erenumab 140 mg was effective in improving MPFID- PI and MPFID- EA compared to placebo and reduction in HIT-6 score at 12 weeks (*p* < 0.001)
**Hirata et al. 2021** [19]	Total: 261 patients Erenumab 70 mg: 130 Placebo: 131	Phase 3, randomized, double-blind, placebo-controlled study of Japanese patients	41 locations in Japan Treatment duration: 24 weeks	Japanese between 20 and 65 years old with a history of chronic or episodic migraine lasting 12 months or more, as defined by ICHD-3, were included in the study	The results of Erenumab 70 mg showed more reduction in MMD compared to placebo at months 4–6 for the prior preventive treatment failure group (*P* = 0.013) and non-prior preventive treatment failure (*P* = 0.012), and ≥ 50% reduction in MMD for prior treatment failure group (*P* = 0.004) but no significant improvement for the no prior failure group
**Diener et al. 2021** [33]	Total: 955 patients Erenumab 70 mg: 317 Erenumab 140 mg: 319 Placebo: 319	Post-hoc analysis of randomized, double-blind, placebo-controlled, Phase 3 study (STRIVE study) [36]	The study occurred from July 2015 until September 5, 2016, at 121 sites in North America, Europe, and Turkey Treatment duration: 24 weeks	Adults between the ages of 18 and 65 who had experienced migraine, with or without aura, for a minimum of 12 months before screening were allowed to join the study	During months 4 through 6 of the DBTP, the changes from baseline in MMD were: a decrease of 1.8 days (22%) with placebo, a decrease of 3.2 days (39%) with Erenumab 70 mg, and a decrease of 3.7 days (44%) with Erenumab 140 mg
**Broessner et al. 2020** [30]	Total: 955 patients Erenumab 70 mg: 317 Erenumab 140 mg: 319 Placebo: 319	Post-hoc for a multicenter, randomized, double-blind, placebo-controlled, parallel-group, phase 3 trial [36]	121 locations in Europe, North America, and Turkey Treatment duration: 24 weeks	Adults aged 18 to 65 who had a history of EM, defined as at least 4 to fewer than 15 migraine days per month and < 15 headache days per month during the 3 months prior to screening and the 4-week baseline phase	At 4 months through 6, the proportions of patients achieving ≥ 50% and ≥ 75% reduction in MMD in Erenumab group was higher than that of the placebo group, with a *P* value < 0.001 for both doses (70 mg and 140 mg)
**Goadsby et al. 2019** [18]	Total: 955 patients Erenumab 70 mg: 317 Erenumab 140 mg: 319 Placebo: 319	Subgroup analysis of a phase 3, multicenter, randomized, double-blind, placebo-controlled, parallel-group (STRIVE) [36]	The study occurred from July 2015 until September 5, 2016, at 121 locations in North America, Europe, and Turkey Treatment duration: 24 weeks	Adults aged 18 to 65 who had a history of EM, defined as at least 4 to fewer than 15 migraine days per month and < 15 headache days per month during the 3 months prior to screening and the 4-week baseline phase	The two doses of Erenumab resulted in a greater reduction in MMD (*P* < 0.05 for 70 mg and *p* < 0.001 for 140 mg) and MSMD compared to placebo in all subgroups. Additionally, more patients reached ≥ 50% and ≥ 75% reduction with any dose of Erenumab compared to placebo
**Brandes et al. 2019** [28]	Total: 667 patients Erenumab 70 mg: 191 Erenumab 140 mg: 190 Placebo: 286	Post-hoc analysis of multicenter, randomized, double-blind, placebo-controlled trial [26]	The study occurred in Europe (Czech Republic, Denmark, Finland, Germany, Norway, Poland, Sweden, and the UK) and North America (Canada and the USA), at 69 clinical research and headache centers Treatment duration: 12 weeks	Patients 18 to 65 years of age with a history of CM were eligible for enrollment. Candidates had to have had a minimum of 15 headache days per month for each of the three months leading up to screening, with a minimum of 8 of those days specifically being migraine days	Results of Erenumab 70 mg and 140 mg showed more patients with ≥ 50% reduction in MMD compared to placebo at 3 months (*p* < 0.001)
**Sakai et al. 2019** [23]	Total: 408 patients Erenumab 70 mg: 135 Erenumab 140 mg: 137 Placebo: 136	Phase 2, randomized, double-blind, placebo-controlled study was conducted in Japan	In Japan at 43 centers with on-site headache specialists Treatment duration: 24 weeks	Adults aged between 20 and 65 years with a history of migraine, ≥ 12 months as per ICHD-3 beta. They also needed to have experienced an average of 4 to < 15 migraine days per month over the three months leading up to the screening	Erenumab 70 mg and 140 mg were effective in improving MMD compared to placebo over 4 to 6 months (*P* < .001), ≥ 50% reduction in MMD (*P* < .001), and MSMD reduction (*P* < .001)
**Reuter et al. 2018 (LIBERTY)** [22]	Total: 246 patients Erenumab 140 mg: 121 Placebo: 125	Phase 3b, randomised, double-blind, placebo-controlled study	59 sites in 16 countries across Europe and Australia Treatment duration: 12 weeks	Adults between 18 and 65 years of age who had episodic migraine, lasting at least 12 months. They need to experience between 4 and 14 migraine days for each month over the 3 months leading up to the screening while experiencing headaches no more than 14 days per month overall. Additionally, they should have previously failed 2 to 4 preventive migraine treatments	Erenumab was effective in improving MMD compared to placebo at 12 weeks (*P* = 0·004), ≥ 50% reduction in MMD (*p* = 0·002) and MSMD reduction (*P* < .001)
**Ashina et al. 2018** [16]	Total: 667 patients Erenumab 70 mg: 191 Erenumab 140 mg: 190 Placebo: 286	Post-hoc analysis of multicenter, randomized, double-blind, placebo-controlled trial [26]	The study occurred in Europe (Czech Republic, Denmark, Finland, Germany, Norway, Poland, Sweden, and the UK) and North America (Canada and the USA), at 69 clinical research and headache centers Treatment duration: 12 weeks	Patients 18 to 65 years of age with a history of CM were eligible for enrollment. Candidates had to have had a minimum of 15 headache days per month for each of the three months leading up to screening, with a minimum of 8 of those days specifically being migraine days	Results of Erenumab 70 mg and 140 mg showed more reduction in MMD compared to placebo at 3 months for prior treatment failure group (*p* < 0.001) and for no prior treatment failure group (70 mg only) (*p* < 0.05), and ≥ 50% reduction in MMD for prior treatment failure group (*p* < 0.001) but no significant effect on the other group
**Buse et al. 2018** [31]	Total: 955 patients Erenumab 70 mg: 317 Erenumab 140 mg: 319 Placebo: 319	Post-hoc for a multicenter, randomized, double-blind, placebo-controlled, parallel-group, phase 3 trial [36]	The study occurred from July 2015 until September 5, 2016, at 121 sites in North America, Europe, and Turkey Treatment duration: 24 weeks	Adults aged 18 to 65 who had a history of EM, defined as at least 4 to fewer than 15 migraine days per month and < 15 headache days per month during the 3 months prior to screening and the 4-week baseline phase	Over months 4–6, Erenumab resulted in more reduction in mMIDAS (*P* < 0.001 for both doses 140 mg and 70 mg) and HIT-6 (*P* < 0.001 for both doses 70 mg and 140 mg) than placebo
**Dodick et al. 2018** [34]	Total: 577 patients Erenumab 70 mg: 286 Placebo: 291	Phase 3, multicenter, randomized, double-blind, placebo-controlled, parallel-group	69 sites across North America and Europe (including Russia). Treatment duration: 12 weeks	Adults aged 18 to 65 who have had EM (characterized by 4 to less than 15 migraine days per month and fewer than 15 headache days per month) with or without aura for at least 12 months prior to the study were eligible to participate	Results of Erenumab showed more reduction in MMD compared to placebo at 3 months (*p* < 0.001), ≥ 50% reduction in MMD (*p* = 0.010), MSMD reduction (*p* = 0.002), and HIT-6 reduction (*p* < 0.001)
**Goadsby et al. 2017 (STRIVE Study)** [36]	Total: 955 patients Erenumab 70 mg: 317 Erenumab 140 mg: 319 Placebo: 319	Phase 3, multicenter, randomized, double-blind, placebo-controlled, parallel-group	The study occurred from July 2015 until September 5, 2016, at 121 sites in North America, Europe, and Turkey Treatment duration: 24 weeks	Adults aged 18 to 65 who had a history of EM, defined as at least 4 to fewer than 15 migraine days per month and < 15 headache days per month during the 3 months prior to screening and the 4-week baseline phase	At month 3, the reduction in MMD was greater for Erenumab group compared to the placebo group (*P* < 0.001) for both doses. Also, the proportion of patients achieving ≥ 50% reduction in MMD was higher for the Erenumab group (*P* < 0.001) for both doses
**Tepper et al. 2017** [26]	Total: 667 patients Erenumab 70 mg: 191 Erenumab 140 mg: 190 Placebo: 286	Phase 2, randomised, double-blind, placebo-controlled	The study occurred in Europe (Czech Republic, Denmark, Finland, Germany, Norway, Poland, Sweden, and the UK) and North America (Canada and the USA), at 69 clinical research and headache centers Treatment duration: 12 weeks	Patients 18 to 65 years of age with a history of CM were eligible for enrollment. Candidates had to have had a minimum of 15 headache days per month for each of the three months leading up to screening, with a minimum of 8 of those days specifically being migraine days	Results of Erenumab 70 mg and 140 mg showed more reduction in MMD compared to placebo at 3 months (*p* < 0.0001), ≥ 50% reduction in MMD (*p* = 0.0001 and *p* < 0.0001, respectively), and MSMD reduction (*p* < 0·0001)
**Hoon et al. 2017** [20]	Single-dose study Total: 12 patients Erenumab 140 mg: 6 Placebo: 6	Phase1, single-center, double-blind, placebo-controlled, sequential-dose-escalation, single-dose study	1 location in Belgium; Treatment duration: 155 days	The study included healthy men and women who are not of childbearing potential—aged 18 to 45 for single-dose trials and 18 to 55 for multiple-dose trials—as well as men and women with migraines aged 18 to 55	The most commonly reported adverse effects were nasopharyngitis, arthralgia, and influenza-like illness
**Sun et al. 2016** [24]	Total: 267 patients Erenumab 70 mg: 107 Placebo: 160	Phase 2, randomised, double-blind, placebo-controlled study	The study occurred at 59 clinical research and headache centers in Europe (Denmark, Finland, Germany, Norway, Sweden, and Portugal) and North America (Canada, USA) and Treatment duration: the open-label extension lasted 12 weeks, and a safety follow-up lasted 12 weeks after the last dose of the drug	Adults aged between 18 to 60 years who had a history of migraine lasting a minimum of 12 months, as defined by ICHD-II, were eligible for enrollment if they experienced between 4 and 14 migraine days for each month, with < 15 total headache days for each month, and if at least 50% of their headache days were migraine days, during each of the 3 months leading up to screening	At month 3, reduction of Erenumab in MMD was significantly greater than that of placebo (*p* = 0·021), and a higher 50% responder rate compared to placebo (*p* = 0·011) was noticed in the Erenumab group. The most notable adverse event was nasopharyngitis

*ICHD* The International Classification of Headache Disorders, *CM* Chronic Migraine, *EM* Episodic Migraine, *MMD* Monthly Migraine Days, *HIT*-6 Headache Impact Test, *MSMD* Monthly Acute Migraine-specific Medication Days, *MPFID- PI* Migraine Physical Function Impact Diary- Physical impairment, *MPFID- EA* Migraine Physical Function Impact Diary- Everyday activities

**Table 2:** Baseline characteristics of study population

Study name	Age (Years), Mean (SD)			Gender, Male, N (%)	History of any preventive treatment use, N (%)			History of any preventive treatment failure, N (%)			Migraine Duration (Years), Mean (SD)		
	**Erenumab 70 mg**	**Erenumab 140 mg**	**Placebo**		**Erenumab 70 mg**	**Erenumab 140 mg**	**Placebo**	**Erenumab 70 mg**	**Erenumab 140 mg**	**Placebo**	**Erenumab 70 mg**	**Erenumab 140 mg**	**Placebo**
**Basedau et al. 2024** [17]	39.1 (12.77)	N/A	41.58 (11.43)	6 (15)	N/A	N/A	N/A	N/A	N/A	N/A	N/A	N/A	N/A
**Filippi et al. 2023** [35]	N/A	48·1 (8·83)	42·6 (11·31)	5 (12.82)	N/A	N/A	N/A	N/A	N/A	N/A	N/A	N/A	N/A
**Yu et al. 2022 (The DRAGON Study)** [29]	41.4 (10.9)	N/A	41.9 (10.9)	103 (18.5)	171 (61.29)	N/A	178 (64.03)	80 (28.67)	N/A	74 (26.62)	18.2 (11.9)	N/A	17.8 (11.4)
**Chowdhury et al. 2022** [32]	34.9 (9.2)	35.4 (7.2)	35.3 (8.9)	74 (21.1)	N/A	N/A	N/A	N/A	N/A	N/A	6.76 (5.96)	6.66 (6.54)	6.87 (5.69)
**Wang et al. 2021 (The EMPOwER Study)** [27]	37.3 (10.0)	37.1 (9.6)	38.0 (10.1)	163(18.11%)	181 (53.6)	119 (53.1)	179 (53.0)	108 (32.0)	72 (32.1)	108 (32.0)	11.1 (9.5)	11.2 (9.7)	12.6 (10.2)
**Takishema et al. 2021** [25]	44.2 (8.5)	N/A	44.6 (9.3)	34(13.02%)	60 (46.2)	N/A	50 (38.2)	59 (45.4)	N/A	58 (44.3)	N/A	N/A	N/A
**Lanteri-Minet et al. 2021** [21]	N/A	44·6 (10·5)	44·2 (10·6)	46 (18.7)	N/A	N/A	N/A	N/A	121 (100)	125 (100)	N/A	N/A	N/A
**Hirata et al. 2021** [19]	44.0 (8.2)	N/A	44.5 (9.6)	25 (14.79)	N/A	N/A	N/A	37 (41.1)	N/A	30 (38.0)	17.71 (11.48)	N/A	18.9 (11.98)
**Diener et al. 2021** [33]	41.1 (11.3)	40.4 (11.1)	41.3 (11.2)	141 (14.76)	133 (42.0)	124 (38.9)	131 (41.1)	127 (40.1)	116 (36.4)	127 (39.8)	19.80 (12.26)	19.72 (12.31)	20.05 (12.18)
**Broessner et al. 2020** [30]	41.1 (11.3)	40.4 (11.1)	41.3 (11.2)	141 (14.76)	133 (42.0)	124 (38.9)	131 (41.1)	127 (40.1)	116 (36.4)	127 (39.8)	N/A	N/A	N/A
**Goadsby et al. 2019** [18]	41.1 (11.3)	40.4 (11.1)	41.3 (11.2)	141 (14.76)	133 (42.0)	124 (38.9)	131 (41.1)	127 (40.1)	116 (36.4)	127 (39.8)	19.80 (12.26)	19.72 (12.31)	20.05 (12.18)
**Brandes et al. 2019** [28]	41.4 (11.3)	42.1 (11.3)	42.1 (11.3)	115 (17.2)	138 (72.3)	138 (72.3)	218 (76.2)	127 (66.5)	126 (66.3)	200 (70.0)	N/A	N/A	N/A
**Sakai et al. 2019** [23]	43 (8.45)	44.25 (7.86)	43 (7.68)	63 (15.44)	80 (59.3)	77 (56.2)	76 (55.9)	43 (48.9)	54 (65.1)	44 (53.0)	N/A	N/A	N/A
**Reuter et al. 2018 (LIBERTY)** [22]	N/A	44·6 (10·5)	44·2 (10·6)	46 (18.7)	N/A	N/A	N/A	N/A	121 (100)	125 (100)	N/A	N/A	N/A
**Ashina et al. 2018** [16]	41.4 (11.3)	42.1 (11.3)	42.1 (11.3)	115 (17.2)	138 (72.3)	138 (72.3)	218 (76.2)	127 (66.5)	126 (66.3)	200 (70.0)	20.7 (12.9)	21.9 (11.8)	22.2 (12.7)
**Buse et al. 2018** [31]	41.1 (11.3)	40.4 (11.1)	41.3 (11.2)	141 (14.76)	133 (42.0)	124 (38.9)	131 (41.1)	127 (40.1)	116 (36.4)	127 (39.8)	19.80 (12.26)	19.72 (12.31)	20.05 (12.18)
**Dodick et al. 2018** [34]	42 (11)	N/A	42 (12)	85 (14.7)	134 (46.9)	N/A	132 (45.4)	117 (87.3)	N/A	115 (87.1)	22 (13)	N/A	20 (12)
**Goadsby et al. 2017 (STRIVE Study)** [36]	41.1 (11.3)	40.4 (11.1)	41.3 (11.2)	141 (14.76)	133 (42.0)	124 (38.9)	131 (41.1)	127 (40.1)	116 (36.4)	127 (39.8)	19.80 (12.26)	19.72 (12.31)	20.05 (12.18)
**Tepper et al. 2017** [26]	41·4 (11·3)	42·9 (11·1)	42·1 (11·3)	115(17.24%)	N/A	N/A	N/A	127 (67%	126 (66%)	200 (70%)	20.7 (12.8)	21.9 (11.8)	22.2 (12.6)
**Hoon et al. 2017** [20]	N/A	23.8 (5.85)	28.5 (11.7)	3 (25)	N/A	N/A	N/A	N/A	N/A	N/A	N/A	N/A	N/A
**Sun et al. 2016** [24]	42·6 (9·9)	N/A	41·4 (10·0)	53(19.85%)	47 (44%)	N/A	66 (41%)	34 (32%)	N/A	60 (38%)	21·5 (11·7)	N/A	20·7 (11·5)

*N/A* Not available, *N* Number, *SD* Standard deviation

### Quality assessment

**Fig. 2:**
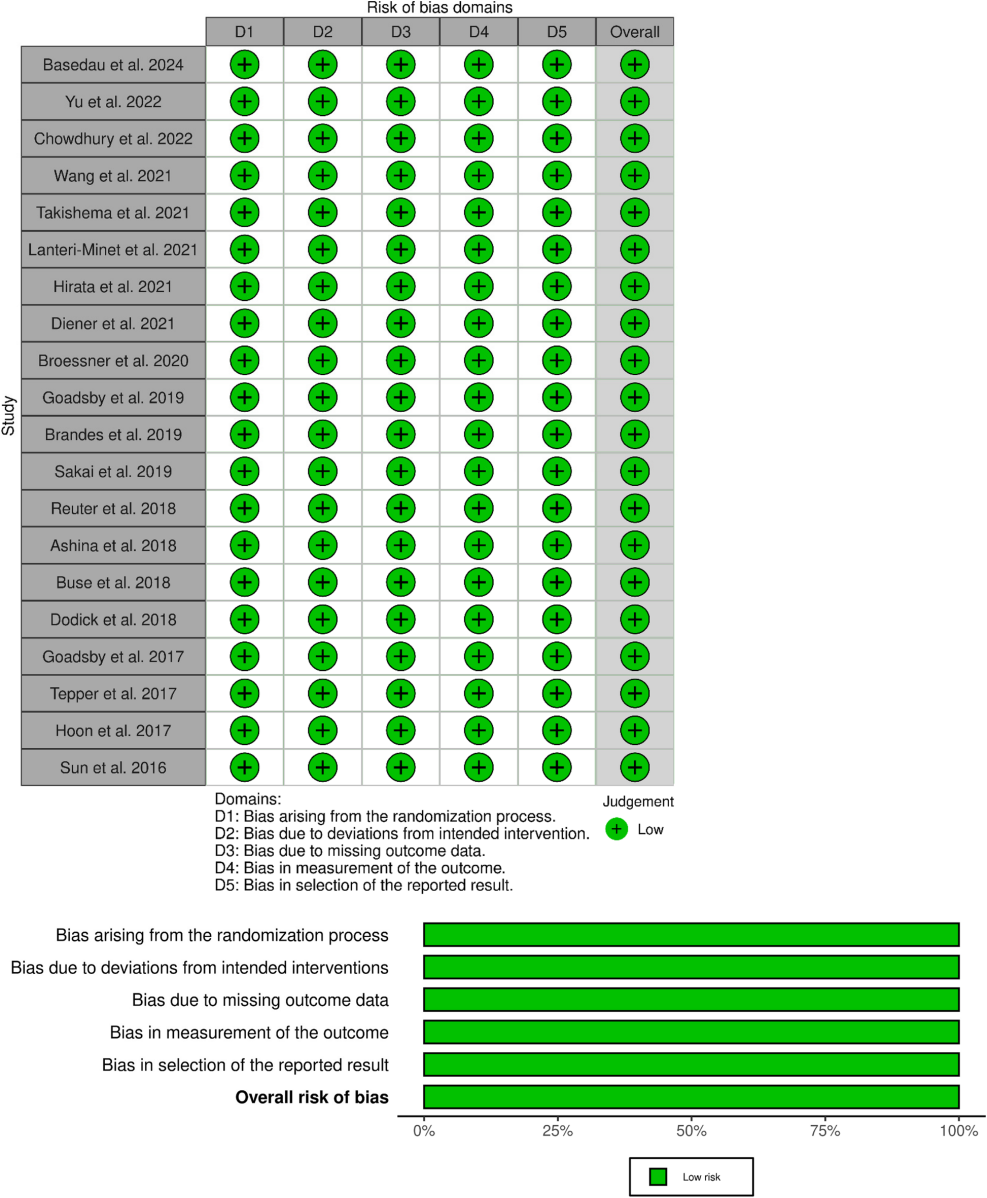
The risk of bias summary and risk of bias graph

The risk of bias assessed by the Cochrane Risk of Bias tool version 2 in all studies is represented in Fig. 2. All the included RCTs have been assigned low risk in all domains and consequently considered to have an overall low risk of bias. Moreover, one study [35] employed a crossover design and was assessed for additional considerations given its different design. It showed some concerns regarding the overall risk of bias, which could be attributed to the unclear reporting of the washout period and carry-over effect details. The risk of bias summary for the crossover study [35] is shown in Supplementary File: Figure (S1).

## Efficacy of Erenumab

### MMD, MSMD, and HIT-6

**Fig. 3:**
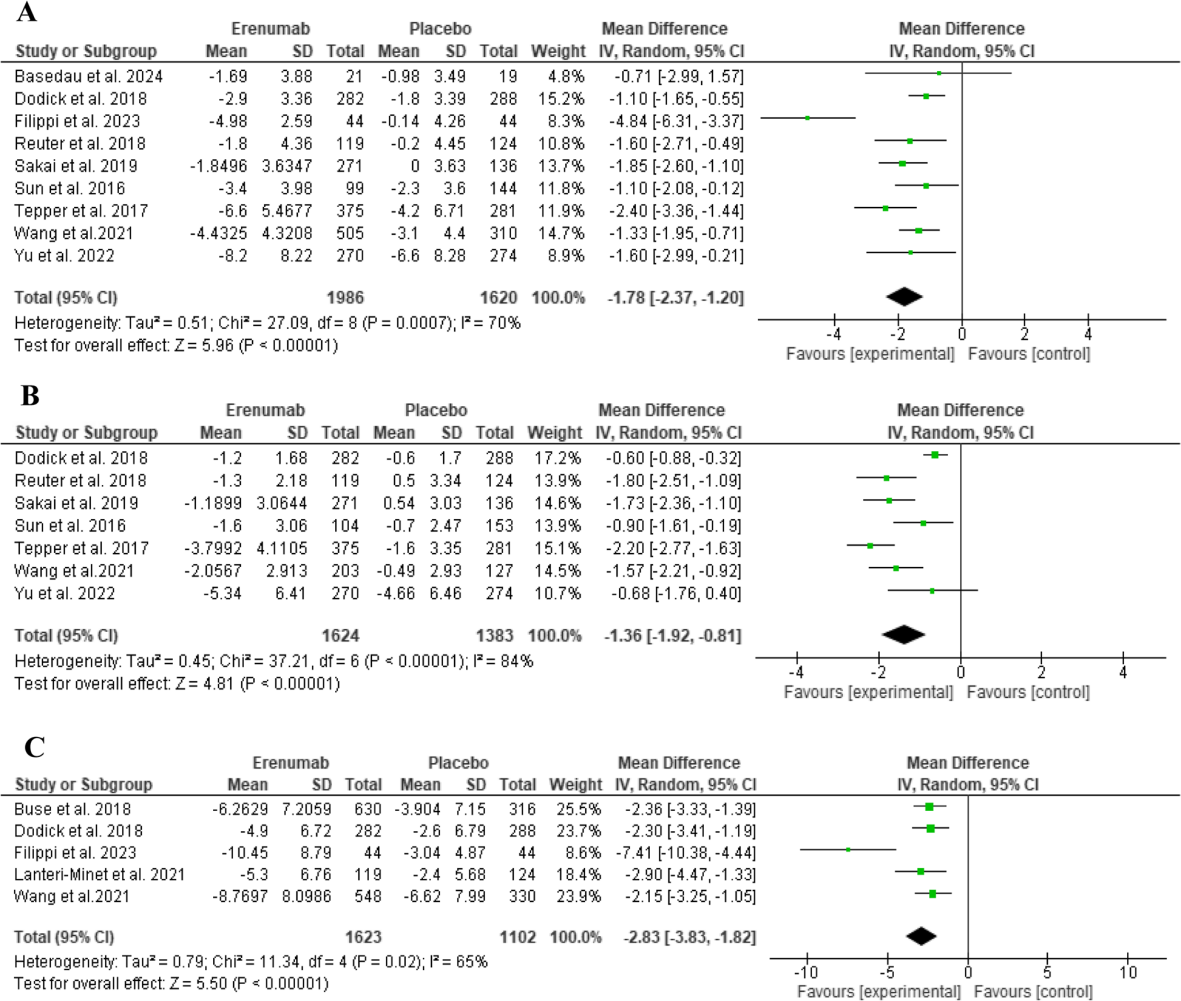
Comparison of Erenumab vs placebo in terms of **A**) MMD, **B**) MSMD, **C**) HIT-6 at three months

Erenumab showed a statistically significant reduction in terms of MMD at three months compared to placebo (MD: -1.78, 95% CI: [-2.37 to -1.20], *P* < 0.00001, as shown in Fig. 3A), and a significant substantial heterogeneity appeared in the analysis (*p* = 0.0007; I^2 = 70%). Similarly, MMD at 4–6 months showed a significant reduction (MD: -1.77, 95% CI: [-2.11 to -1.43], *P* < 0.00001, as shown in Supplementary File: Figure (S2)). However, no heterogeneity was evident in the analysis (*p* = 0.70; I^2 = 0%). Both MSMD at three months and 4–6 months showed statistically significant improvement in favour of Erenumab compared to placebo (MD: -1.36, 95% CI: [-1.92 to -0.81], *P* < 0.00001, as shown in Fig. 3B) and (MD: -1.56, 95% CI: [-2.08 to -1.03], *P* < 0.00001, as shown in Supplementary File: Figure (S2)), respectively. Heterogeneity was significant in both (*p* < 0.00001; I^2 = 84%) and (*p* = 0.02; I^2 = 69%), respectively. HIT-6 at three months exhibited a statistically significant reduction favouring Erenumab (MD: -2.83, 95% CI: [-3.83 to -1.82], *P* < 0.00001, as shown in Fig. 3C), and showed significant heterogeneity (*p* = 0.02; I^2 = 65%).

###  ≥ 50%, ≥ 75%, and 100% reduction from baseline in MMD

**Fig. 4:**
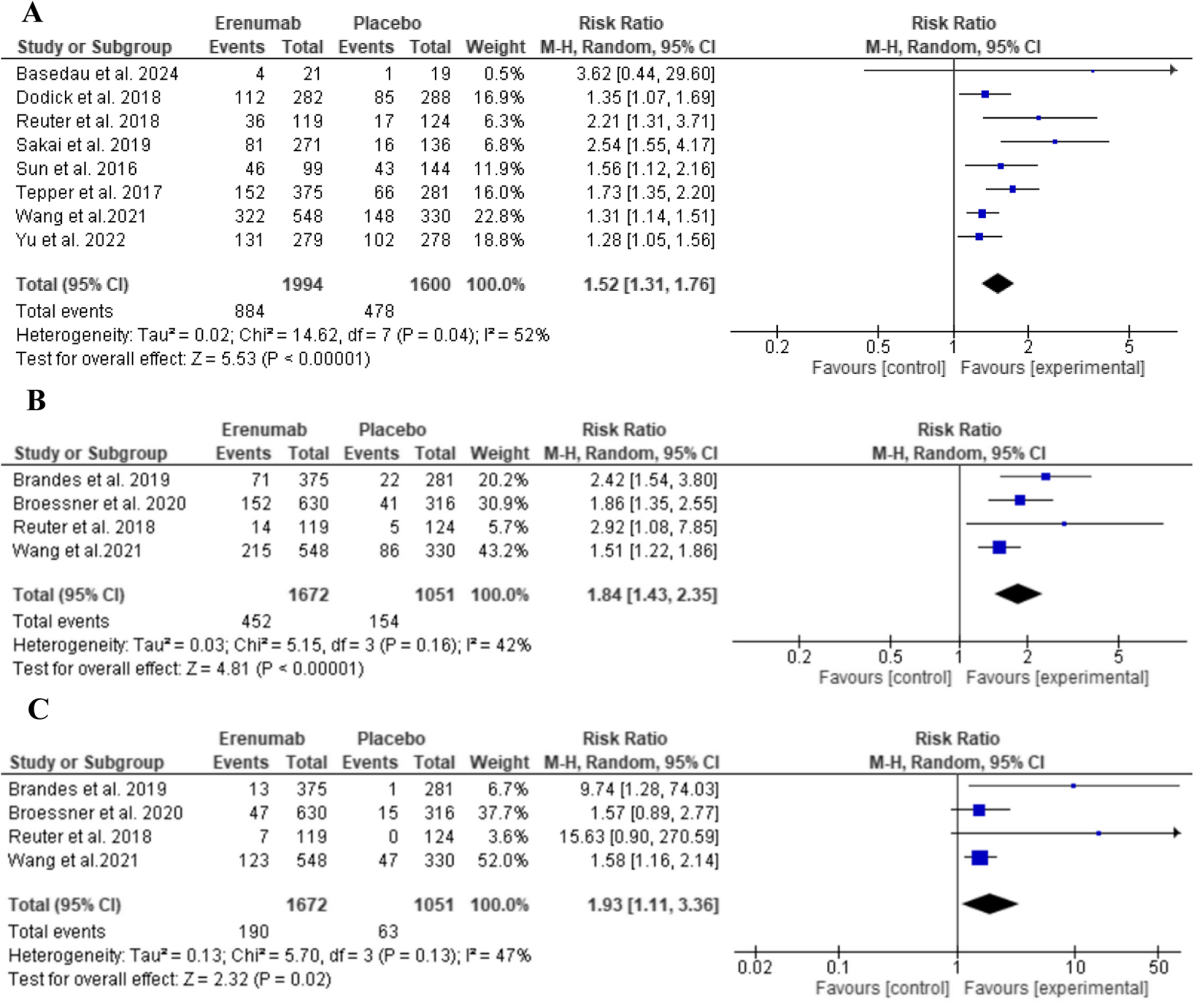
Comparison of Erenumab vs placebo in terms of **A**) ≥ 50%, **B**) ≥ 75%, **C**) 100% reduction from baseline in MMD at three months

The drug showed statistically significant improvements in all responder rates, where significantly more subjects reached ≥ 50% reduction in MMD at three months (RR: 1.52, 95% CI: [1.31 to 1.76], *P* < 0.00001, as shown in Fig. 4A) and at 4–6 months (RR: 2.03, 95% CI: [1.53 to 2.70], *P* < 0.00001, as shown in Supplementary File: Figure (S2)). Similarly, 75% and ≥ 100% reductions from baseline in MMD at three months were significant compared to placebo (RR: 1.84, 95% CI: [1.43 to 2.35], *P* < 0.00001, as shown in Fig. 4B) and (RR: 1.93, 95% CI: [1.11 to 3.36], *P* = 0.02, as shown in Fig. 4C), respectively. ≥ 50% responder rate outcome at three months showed significant moderate heterogeneity (*p* = 0.04; I^2 = 52%).

### MPFID-PI, MPID-EA, and mMIDAS at three months

**Fig. 5:**
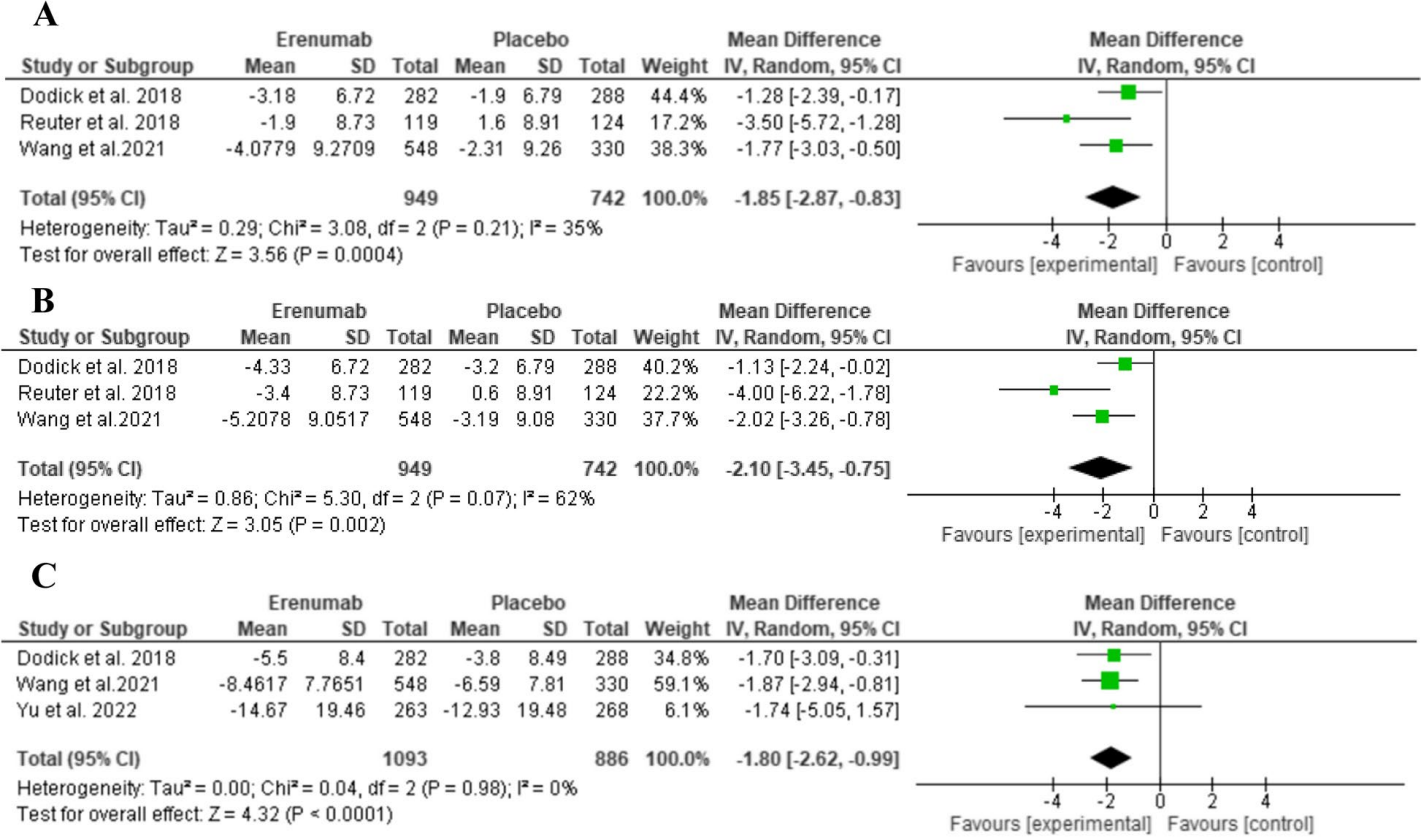
Comparison of Erenumab vs placebo in terms of **A**) MPFID-PI, **B**)MPID-EA, **C**) mMIDAS at three months

Each of MPFID-PI, MPID-EA, and mMIDAS showed a statistically significant reduction favouring Erenumab compared to placebo, (MD: -1.85, 95% CI: [-2.87 to -0.83], *P* = 0.0004, as shown in Fig. 5A), (MD: -2.10, 95% CI: [-3.45 to -0.75], *P* = 0.002, as shown in Fig. 5B), and (MD: -1.80, 95% CI: [-2.62 to -0.99], *P* < 0.00001, as shown in Fig. 5C), respectively.

## Sensitivity analysis

Sensitivity analysis by removing one study at a time has been conducted to investigate sources of heterogeneity and test the robustness of the analysis in our primary outcomes. In both MMD (*p* = 0.0007; I^2 = 70%) and HIT-6 (*p* = 0.02; I^2 = 65%) at three months, the substantial heterogeneity was resolved by excluding Filippi et al. 2023 [35], yielding homogenous results (*p* = 0.38; I^2 = 7%) and (*p* = 0.90; I^2 = 0%), respectively, as shown in Supplementary File: Figure (S3).

Regarding MSMD at three months, heterogeneity (*P* < 0.00001; I^2 = 84%) was resolved by excluding both Dodick et al. 2018 [34] and Tepper et al. 2017 [26] (*P* = 0.19; I^2 = 35%). Moreover, the heterogeneity in ≥ 50% responder rate outcome at three months (*p* = 0.04; I^2 = 52%) was resolved by excluding Sakai et al. [23] (*p* = 0.18; I^2 = 33%), as shown in Supplementary File: Figure (S3). No single study exclusion affected the overall significance of any of the study`s outcomes, ensuring robust results.

## Subgroup analysis

We conducted subgroup analysis according to multiple criteria not only to investigate sources of heterogeneity but also to provide estimates of treatment effects for clinically relevant subgroups of patients. According to the status of previous failures, in all three primary outcomes, MMD, MSMD, and ≥ 50% reduction in MMD, the test for subgroup differences suggested that there was a statistically significant subgroup difference, *p* = 0.02, *p* = 0.010, *p* = 0.0007, respectively, as shown in Table 3. The presence of prior failure significantly modified the response of Erenumab compared to placebo. The treatment effect favoured Erenumab over placebo in both subgroups; however, it was significantly higher for the subgroup with prior failures indicating a quantitative subgroup effect. Although there was a significant difference between subgroups, an uneven distribution of trials and participants between subgroups existed. The forest plots are shown in Supplementary File: Figure (S4).

**Table 3:** Summary of the subgroup analysis for the primary outcomes

Category	Outcomes	Subgroups	No. of studies	Pooled effect estimate [95% CI]	Subgroup difference (*P*-value, I^2)
**Presence of previous failures**	MMD 3 months	No failure	3	-1.24 [-1.82: -0.67]	*P* = 0.02*, I^2 = 80.3%
		Prior failure	5	-2.58 [-3.60: -1.57]	
	MSMD 3 months	No failure	3	-0.92 [-1.25: -0.59]	*P* = 0.010*, I^2 = 85.1%
		Prior failure	4	-1.93 [-2.62: -1.24]	
	≥ 50% reduction in MMD	No failure	3	1.38 [1.16: 1.65]	*P* = 0.0007*, I^2 = 91.4%
		Prior failure	4	2.28 [1.82: 2.85]	
**Type of migraine**	MMD 3 months	Episodic	6	-1.80 [-2.53: -1.06]	*P* = 0.53, I^2 = 0%
		Chronic	2	-2.14 [-2.93: -1.35]	
	≥ 50% reduction in MMD	Episodic	5	1.57 [1.27: 1.93]	*P* = 0.74, I^2 = 0%
		Chronic	2	1.47 [1.10: 1.98]	
**Dose of Erenumab**	MMD 3 months	70 mg	7	-1.39 [-1.77: -1.01]	*P* = 0.07, I^2 = 69.8%
		140 mg	5	-2.36 [-3.34: -1.38]	
	MSMD 3 months	70 mg	6	-1.18 [-1.69: -0.67]	*P* = 0.01*, I^2 = 84%
		140 mg	4	-1.99 [-2.35: -1.62]	
	≥ 50% reduction in MMD	70 mg	8	1.47 [1.28: 1.68]	*P* = 0.15, I^2 = 52.7%
		140 mg	5	1.57 [1.40: 1.76]	

In terms of different doses of Erenumab, only the MSMD outcome revealed a statistically significant test for subgroup difference (*p* = 0.01) with a quantitative subgroup effect showing a more significant improvement favouring the 140 mg dose over the 70 mg compared to placebo, as shown in Table 3. The forest plots demonstrating subgroups of different doses are shown in Supplementary File: Figure (S5). Regarding the type of migraine, the test for subgroup differences indicated that there was no statistically significant subgroup difference in either MMD or ≥ 50% reduction in MMD (*p* = 0.48) and (*p* = 0.69), respectively, as shown in Table 3. This finding suggested that the type of migraine does not influence the effect of Erenumab compared to placebo. However, a smaller number of trials and participants contributed data to the chronic migraine subgroup (two studies) than to the episodic migraine subgroup (six studies in MMD and five in ≥ 50% reduction in MMD), proposing a possibility that the analysis may not be able to detect subgroup differences. The forest plots of subgrouping according to the type of migraine are shown in Supplementary File: Figure (S6).

### Publication bias

**Fig. 6:**
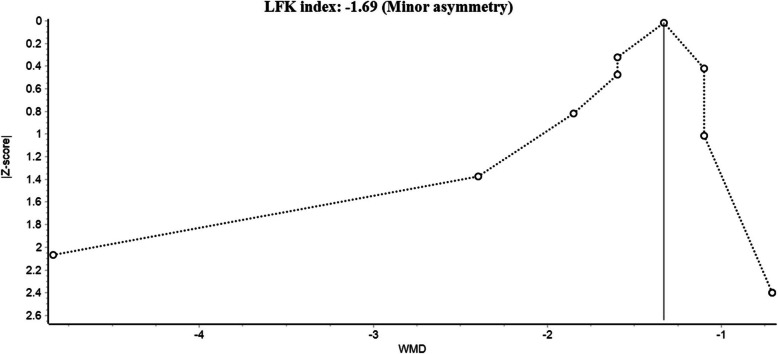
Doi plot and LFK index for MMD at 3 months

Regarding publication bias, the DOI plot of MMD at 3 months showed minor asymmetry with an LFK index of -1.69, as shown in Fig. 6, suggesting possible publication bias. MSMD, HIT-6, and ≥ 50% reduction in MMD at 3 months showed major asymmetry in the DOI plots with LFK indices of -3.51, -2.95, and 5.92, respectively, indicating potential publication bias. The DOI plots of MSMD, HIT-6, and ≥ 50% reduction in MMD at 3 months are shown in Supplementary File: Figure (S7). Furthermore, funnel plots for MMD, HIT-6, MSMD, and ≥ 50% reduction in MMD at 3 months are shown in Supplementary File: Figures (S8) and (S9).

### Safety of erenumab

Erenumab showed an acceptable safety profile compared to placebo. The intervention resulted in a significantly higher incidence of only constipation compared to the placebo (RR: 2.53, 95% CI: [1.60 to 4.02], *P* < 0.0001). Although nasopharyngitis and upper respiratory tract infection were the most reported adverse effects in both the placebo and Erenumab groups, they were not statistically significant. A summary of the adverse events is displayed in Table 4. All the forest plots demonstrating the adverse effects are represented in Supplementary File: Figures (S10)—(S14).

**Table 4:** Summary of the adverse events

Adverse events	Number of studies	Erenumab		Placebo		Risk Ratio (95%CI)	*P* Value
		**Events**	**Total**	**Events**	**Total**		
**Any adverse event**	12	1475	2914	1113	2219	1.02 [0.94, 1.11]	0.63
**Any serious adverse event**	11	45	2809	37	2183	0.90 [0.58, 1.39]	0.62
**Any adverse event leading to treatment discontinuation**	11	30	2908	19	2183	1.07 [0.59, 1.92]	0.82
**Nasopharyngitis**	10	243	2765	182	2053	0.93 [0.77, 1.12]	0.45
**Upper respiratory tract infection**	8	99	2365	67	1786	1.09 [0.80, 1.48]	0.60
**Constipation**	7	102	2534	28	1770	2.53 [1.60, 4.02]	< 0.0001*
**Nausea**	5	34	1626	29	1166	0.90 [0.54, 1.50]	0.69
**Urinary tract infection**	4	14	1520	8	1013	0.90 [0.40, 2.06]	0.81
**Back Pain**	4	25	988	19	727	1.05 [0.57, 1.93]	0.88
**Influenza**	3	24	1022	21	761	0.96 [0.52, 1.77]	0.90
**Injection site pain**	4	49	1413	23	1014	1.76 [0.96, 3.25]	0.07
**Abdominal pain**	4	10	882	2	547	2.05 [0.54, 7.72]	0.29
**Vomiting**	3	3	610	2	411	1.00 [0.20, 5.03]	1.00
**Diarrhea**	4	9	468	7	413	1.12 [0.22, 5.60]	0.89
**Migraine**	4	24	1400	23	1043	0.88 [0.38, 2.08]	0.78
**Arthralgia**	3	17	745	11	478	1.05 [0.34, 3.23]	0.94
**Fatigue**	4	30	1141	19	885	1.26 [0.71, 2.22]	0.43
**Gastroenteritis**	4	12	967	9	608	0.90 [0.39, 2.08]	0.81

^*^significant difference between groups (*p*-value less than 0.05)

### Quality of the evidence

The certainty of evidence regarding Erenumab efficacy in the most important and relevant outcomes was assessed using GRADE. All the outcomes at three months, including MMD, MSMD, HIT-6, and ≥ 50% reduction in MMD, were downgraded in two domains: inconsistency and the presence of suspected publication bias. Therefore, this downgrade resulted in a low overall certainty of evidence regarding the aforementioned four outcomes. A summary of the findings and a GRADE evaluation of the outcomes are shown in Table 5.

**Table 5:** Quality of evidence and summary of findings

Certainty assessment							Summary of findings				
**Outcomes/** **number of patients (number of studies)**	**Risk of bias**	**Inconsistency**	**Indirectness**	**Imprecision**	**Publication bias**	**Overall certainty of evidence**	**Study event rates (%)**		**Relative effect** **(95% CI)**	**Anticipated absolute effects**	
							**With Placebo**	**With Erenumab**		**Risk with Placebo**	**Risk difference with Erenumab**
**MMD at 3 months**											
3606 (9 RCTs)	not serious	serious^a^	not serious	not serious	publication bias suspected^b^	⨁⨁◯◯ Low	1620	1986	-	1620	MD **1.78 days per month lower** (2.37 lower to 1.2 lower)
**MSMD at 3 months**											
3007 (7 RCTs)	not serious	serious^a^	not serious	not serious	publication bias suspected^b^	⨁⨁◯◯ Low	1383	1624	-	1383	MD **1.36 days per month lower** (1.92 lower to 0.81 lower)
**HIT-6 at 3 months**											
2725 (5 RCTs)	not serious	serious^a^	not serious	not serious	publication bias suspected^b^	⨁⨁◯◯ Low	1102	1623	-	1102	MD **2.83 points lower** (3.83 lower to 1.82 lower)
** ≥ 50% reduction in MMD at 3 months**											
3594 (8 RCTs)	not serious	serious^a^	not serious	not serious	publication bias suspected^b^	⨁⨁◯◯ Low	478/1600 (29.9%)	884/1994 (44.3%)	**RR 1.52** (1.31 to 1.76)	478/1600 (29.9%)	**155 more per 1,000** (from 93 to 227 more)

## Discussion

In this meta-analysis, we assessed the efficacy and safety of Erenumab in patients with episodic and chronic migraine. Erenumab resulted in a significant reduction in MMD and HIT-6 compared to placebo. However, there was considerable heterogeneity, with ${I}^{2}$ =70% for MMD and 65% for HIT-6TM, possibly due to several reasons. First and most importantly, the crossover design utilized in the study by Filippi et al. [35] (where patients were randomly assigned to receive either Erenumab or placebo for 12 weeks, then switched to the other treatment for another 12 weeks) raised some concerns that it might contribute to this heterogeneity. Filippi et al. reported they observed that the impact of Erenumab on MMD at week 16 persisted in patients who were randomized to the treatment sequence of Erenumab followed by placebo (who discontinued the drug at week 12). However, there was no evidence that the carry-over effect persisted up to week 24 when data from the second period were included in our analysis. Besides, Jenssen et al. addressed the crossover design in migraine preventive treatment in four RCTs and detected no carry-over effect in migraine patients [57]. However, it is noteworthy that Filippi et al. [35] included only patients who had failed two or more previous migraine preventives, which could be a plausible cause for heterogeneity. This hypothesis was confirmed by the resolution of heterogeneity when conducting a leave-one-out analysis for the study by Filippi et al. [35].

Although there was a significant decline in MSMD with Erenumab, substantial heterogeneity was evident (${I}^{2}$=84%), which was resolved by excluding Dodick et al. [34] and Tepper et al. [26] Additionally, excluding Sakai et al. [23] resolved the heterogeneity with ≥ 50% response rate outcome. Differences in the eligibility criteria of each study regarding the use of concurrent or prior migraine preventive treatment might account for this heterogeneity. For instance, Tepper et al. [26] prevented migraine preventive drugs during the study and two months before the baseline. Sakai et al. [23] and Dodick et al. [34] allowed using one preventive drug as long as the dose did not differ from two months before. Moreover, Reuter et al. [22] required patients to have failed previous treatment with 2–4 preventive medications.

Our subgroup analysis revealed that patients with prior failures to preventive treatments exhibited a higher response to Erenumab compared to those without such a history. This enhanced treatment effect appears to be influenced, in part, by a lower placebo response in this subgroup, particularly concerning migraine frequency-related endpoints such as change in MMD, ≥ 50%, and ≥ 75% response rates [16, 18, 22]. This observation is consistent across multiple studies, where a reduced placebo effect has been linked to lower expectations among patients who have unsuccessfully tried several preventive medications [16, 18, 22]. Moreover, the placebo response in treatment-naive patients tends to be higher, which may dilute the apparent efficacy of active treatments in such populations [22, 26, 34, 36]. However, the uniform placebo response observed in the MSMD endpoint suggests that the influence of prior treatment failures may vary across different outcome measures [18]. Overall, these findings underscore the importance of considering prior treatment history in the design and interpretation of migraine trials, as including patients with failed prior treatments might reduce placebo effects and provide a better assessment of the treatment's true efficacy. Further research is warranted to explore the mechanisms underlying these differences and to optimize treatment strategies for this challenging patient population. The aforementioned results are consistent with the recommendations of the European Headache Federation guidelines [58] and the position statement of the American Headache Society [59], which suggest using a CGRP monoclonal antibody in patients who have failed two or more preventive treatments. Besides, subgroup analysis by doses revealed no significant difference between the two doses (70 mg and 140 mg) in primary outcomes, except for MSMD, in which Erenumab 140 mg exhibited significantly higher response than the 70 mg dose. This finding aligns with Gui MM et al., who also reported that the 140 mg dose was more effective than the 70 mg dose specifically for MSMD [60].

Regarding physical function in migraine patients, Yang et al. reported no significant difference between the placebo and Erenumab groups in the change of MPFID-EA and MPFID-PI scores from baseline [61]. In contrast, our analysis showed that Erenumab led to a statistically significant greater reduction in both scores compared to placebo. These findings indicate that Erenumab not only alleviates migraine symptoms but also improves physical function and daily activity performance.

In terms of safety, although the most common adverse events were nasopharyngitis and upper respiratory tract infection, there was no statistically significant difference between both groups. Most importantly, Erenumab showed a statistically significant increase in rates of constipation compared to placebo (RR: 2.53, *P* < 0.0001). These higher rates of constipation could be explained by the crucial role CGRP plays in regulating gastrointestinal motor activity, thus affecting intestinal transit, propulsion, and secretion [62, 63]. Lampl et al. [64] found that both doses of Erenumab (70 mg and 140 mg) demonstrated a safety profile comparable to that of a placebo for patients with episodic or chronic migraine. This finding was consistent across all age groups, including those aged 50 and older, with no increase in adverse events noted in the older age group. In contrast, many standard oral preventive treatments are used cautiously in older adults due to poor safety and tolerability profiles. Therefore, Erenumab emerges as a viable, well-tolerated, and effective alternative for patients of all ages [64].

To the best of our knowledge, this is the largest and most comprehensive meta-analysis evaluating the efficacy and safety of Erenumab for migraine patients. Notably, our meta-analysis is the first to conduct a subgroup analysis based on prior migraine-preventive treatment failure status. We performed two other subgroup analyses according to the type of migraine and doses. Besides, we conducted sensitivity analyses to detect the source of heterogeneity found in primary outcomes. Furthermore, to assess the quality of the evidence, we utilized the GRADE system. Given the small number of studies per outcome, we employed Doi plots and the LFK index to evaluate publication bias. Nevertheless, there are inevitable limitations. First, the small and unequal distribution of sample sizes and studies in the subgroup analysis might cause imbalance and affect the overall analysis, potentially leading to biased or less generalizable results for specific subgroups. Additionally, variability in study protocols posed another challenge, as some studies allowed participants to use a concomitant migraine-preventive treatment during the study, while others prohibited using any. This inconsistency could have impacted the overall assessment of Erenumab’s efficacy, potentially underestimating or overestimating its effects. Due to the unavailability of separate data for patients who used concomitant preventive treatment and those who did not, we could not perform a subgroup analysis. Furthermore, the different definitions of a "migraine day" among studies represented another limitation. Most studies defined a migraine day as any calendar day on which the patient experienced a qualified migraine lasting for ≥ 30 min with either ≥ 2 pain features or ≥ 1 associated non-pain feature. However, four studies [19, 25, 26, 29] required qualified migraine to have lasted for at least 4 h to be considered a migraine day.

Therefore, we recommend that future research focus on carrying out more RCTs comparing patients with migraine who have failed previous preventive treatment with those who have not failed any prior treatment. Such studies could help provide better insights into the enhanced effectiveness of Erenumab in the non-failure group. Furthermore, it would be valuable to conduct studies evaluating the efficacy of Erenumab when administered concomitantly with other migraine-preventive treatments. Additionally, long-duration RCTs investigating the formation of anti-Erenumab antibodies and their potential impact on clinical improvement are necessary.

## Conclusion

In conclusion, this meta-analysis demonstrated that Erenumab significantly reduced the frequency of migraine attacks, the number of days on which migraine patients require medication, and physical impairment scores. Erenumab was more effective in patients with prior preventive treatment failures compared to patients with no prior failure. There was no significant difference in Erenumab`s response between episodic and chronic migraine patients. Furthermore, Erenumab administered at a dose of 140 mg showed superior efficacy in MSMD reduction compared to the 70 mg dose, though no significant differences were evident in MMD or ≥ 50% response rates. The safety profile of Erenumab was comparable to that of the placebo, with constipation being the only significant adverse event for Erenumab. Overall, Erenumab is a safe and effective treatment for migraine. However, long-term RCTs comparing Erenumab with other preventive migraine treatments, including further investigations focused on patient groups with a history of prior treatment failures, are required.

## Supplementary Information

Supplementary Material 1: Figure (S1) The risk of bias summary for the crossover study (Filippi et al.). Figure (S2) Comparison of Erenumab vs placebo at 4-6 months in terms of (A) MMD, (B) MSMD, (C) 50% reduction in MMD. Figure (S3) Sensitivity Analysis of primary outcomes at 3 months. (A) MMD by excluding Filippi et al., (B) HIT-6 by excluding Filippi et al., (C) MSMD by excluding Dodick et al. and Tepper et al., (D) 50% reduction in MMD by excluding Sakai et al. Figure (S4) Subgroup analysis based on prior preventive treatment failure status (Prior failure versus No failure). (A) MMD at 3 months, (B) MSMD at 3 months (C) 50% reduction in MMD at 3 months. Figure (S5) Subgroup analysis based on doses (Erenumab 70mg versus Erenumab 140mg). (A) MMD at 3 months, (B) MSMD at 3 months, (C) 50% reduction in MMD at 3 months. Figure (S6) Subgroup analysis based on type of migraine (Episodic versus Chronic). (A) MMD at 3 months, (B) 50% reduction in MMD at 3 months. Figure (S7) Doi plot and LFK index for (A) HIT-6 at 3 months, (B) MSMD at 3 months, (C) 50% reduction in MMD at 3 months. Figure (S8) Funnel plots for (A) MMD 3 months, (B) HIT-6 at 3 months. Figure (S9) Funnel plots for (A) MSMD at 3 months, (B) 50% reduction in MMD at 3 months. Figure (S10) Adverse Events. (A) Any adverse event, (B) Any serious adverse event (C) Any adverse event leading to treatment discontinuation. Figure (S11) Adverse Events (A) Nasopharyngitis (B) Upper respiratory tract infection (C) Constipation. Figure (S12) Adverse Events (A) Nausea, (B) Urinary Tract Infection, (C) Back pain, (D) Influenza. Figure (S13) Adverse Events (A) Injection site pain, (B) Abdominal pain, (C) Vomiting, (D) Diarrhea. Figure (S14) Adverse Events (A) Migraine, (B) Arthralgia, (C) Fatigue, (D) Gastroenteritis.

## Data Availability

Data is provided within the manuscript or supplementary information files.

## Abbreviations

CM: Chronic Migraine
EM: Episodic Migraine
CGRP: Calcitonin Gene-related Peptide
MMD: Monthly Migraine Days
HIT-6: Headache Impact Test
MSMD: Monthly Acute Migraine-specific Medication Days
MPFID- PI: Migraine Physical Function Impact Diary- Physical impairment
MPFID- EA: Migraine Physical Function Impact Diary- Everyday activities
mMIDAS: Modified Migraine Disability Assessment
ICHD: The International Classification of Headache Disorders
RoB: Cochrane risk-of-bias tool for randomized trials
PRISMA: Preferred Reporting Items for Systematic Reviews and Meta-Analyses
RevMan: Review Manager software
GRADE: Grading of Recommendations, Assessment, Development, and Evaluations
MD: Mean Difference
CI: Confidence Interval
RR: Risk Ratio
