# Supplementary material for: Unveiling the efficacy and safety of Erenumab, a monoclonal antibody targeting calcitonin gene-related peptide (CGRP) receptor, in patients with chronic and episodic migraine: a GRADE-assessed systematic review and meta-analysis of randomized clinical trials with subgroup analysis
Source: Head Face Med. 2025 Mar 26;21:19. doi: 10.1186/s13005-025-00494-w (PMC11938773; doi:10.1186/s13005-025-00494-w)
Supplement: Supplementary file 1 — Supplementary Material 1: Figure (S1) The risk of bias summary for the crossover study (Filippi et al.). Figure (S2) Comparison of Erenumab vs placebo at 4-6 months in terms of (A) MMD, (B) MSMD, (C) 50% reduction in MMD. Figure (S3) Sensitivity Analysis of primary outcomes at 3 months. (A) MMD by excluding Filippi et al., (B) HIT-6 by excluding Filippi et al., (C) MSMD by excluding Dodick et al. and Tepper et al., (D) 50% reduction in MMD by excluding Sakai et al. Figure (S4) Subgroup analysis based on prior preventive treatment failure status (Prior failure versus No failure). (A) MMD at 3 months, (B) MSMD at 3 months (C) 50% reduction in MMD at 3 months. Figure (S5) Subgroup analysis based on doses (Erenumab 70mg versus Erenumab 140mg). (A) MMD at 3 months, (B) MSMD at 3 months, (C) 50% reduction in MMD at 3 months. Figure (S6) Subgroup analysis based on type of migraine (Episodic versus Chronic). (A) MMD at 3 months, (B) 50% reduction in MMD at 3 months. Figure (S7) Doi plot and LFK index for (A) HIT-6 at 3 months, (B) MSMD at 3 months, (C) 50% reduction in MMD at 3 months. Figure (S8) Funnel plots for (A) MMD 3 months, (B) HIT-6 at 3 months. Figure (S9) Funnel plots for (A) MSMD at 3 months, (B) 50% reduction in MMD at 3 months. Figure (S10) Adverse Events. (A) Any adverse event, (B) Any serious adverse event (C) Any adverse event leading to treatment discontinuation. Figure (S11) Adverse Events (A) Nasopharyngitis (B) Upper respiratory tract infection (C) Constipation. Figure (S12) Adverse Events (A) Nausea, (B) Urinary Tract Infection, (C) Back pain, (D) Influenza. Figure (S13) Adverse Events (A) Injection site pain, (B) Abdominal pain, (C) Vomiting, (D) Diarrhea. Figure (S14) Adverse Events (A) Migraine, (B) Arthralgia, (C) Fatigue, (D) Gastroenteritis. [file 13005_2025_494_MOESM1_ESM.docx]

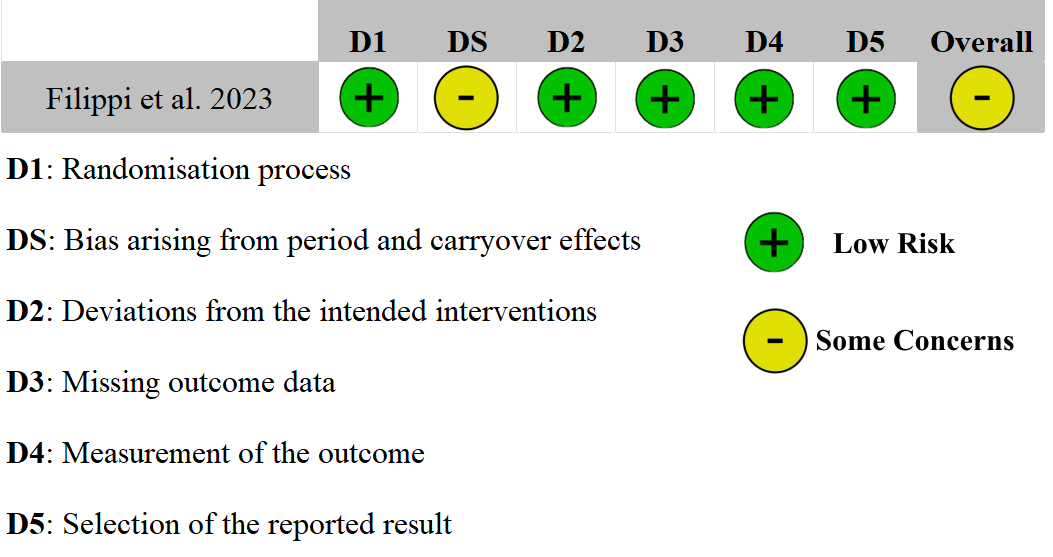


**Figure (S1)** The risk of bias summary for the crossover study (Filippi et al.)

**(A)**

**(C)**


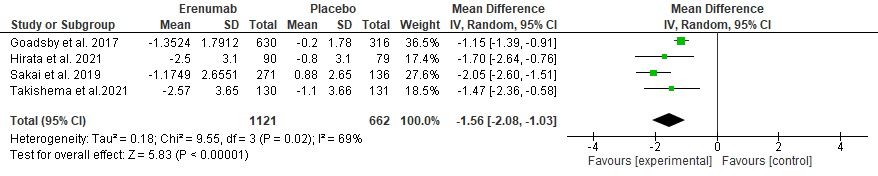


**(B)**


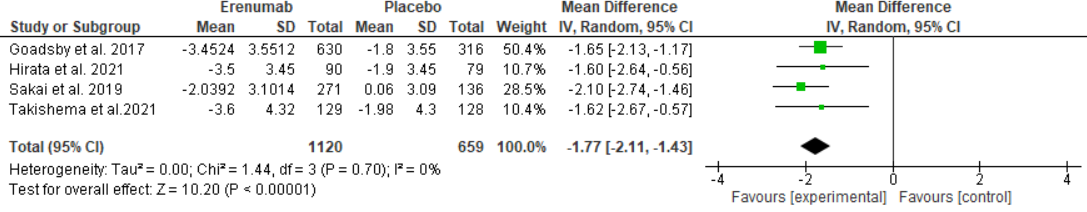

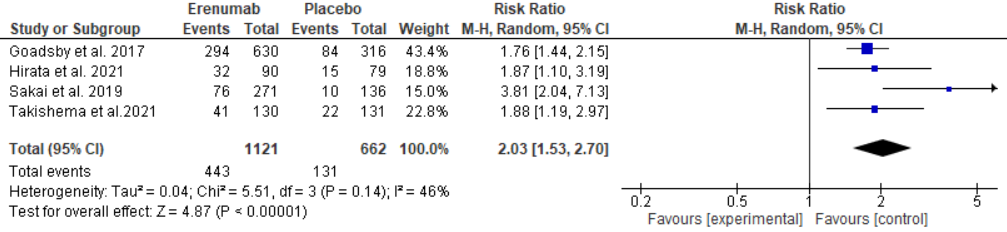


**Figure (S2)** Comparison of Erenumab vs placebo at 4-6 months in terms of **(A)** MMD, **(B)** MSMD, **(C)** 50% reduction in MMD


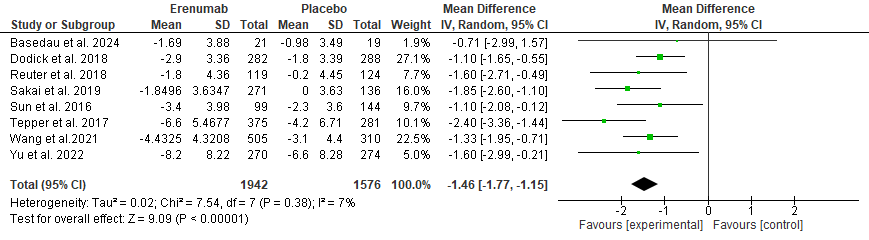

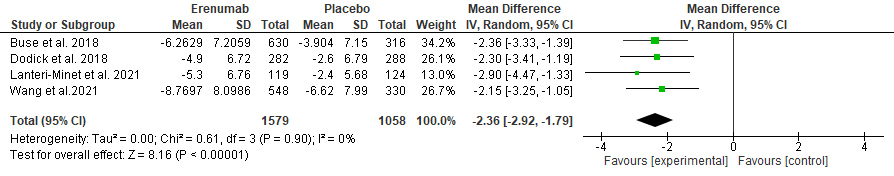

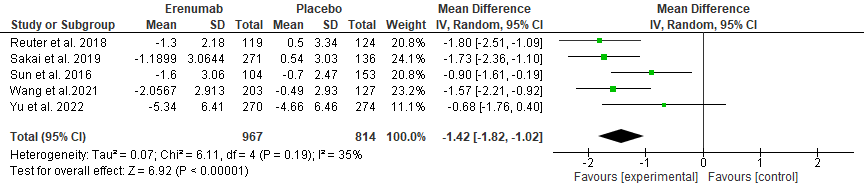

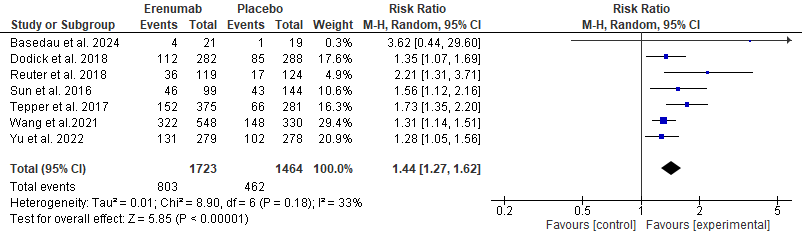


**(A)**

**(B)**

**(C)**

**(D)**

**Figure (S3)** Sensitivity Analysis of primary outcomes at 3 months. **(A)** MMD by excluding Filippi et al., **(B)** HIT-6 by excluding Filippi et al., **(C)** MSMD by excluding Dodick et al. and Tepper et al.**, (D)** 50% reduction in MMD by excluding Sakai et al.


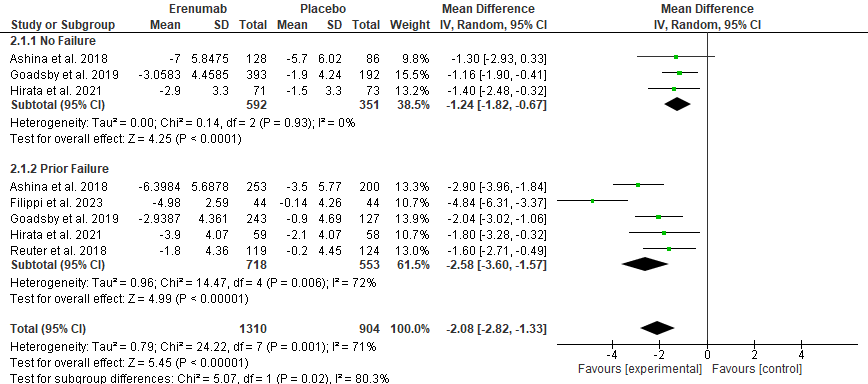


**(A)**

**(B)**


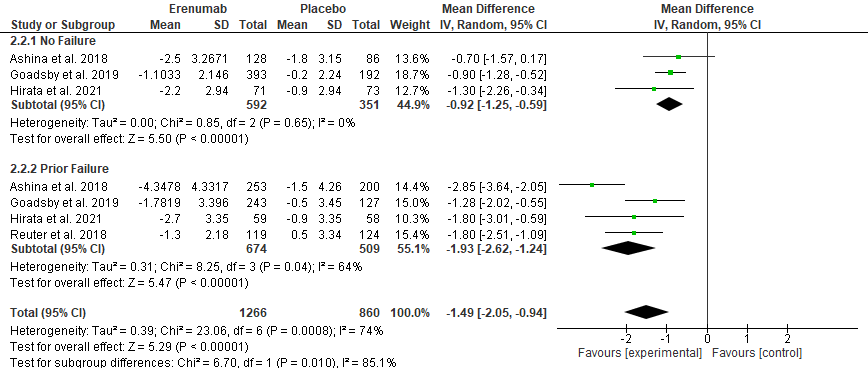

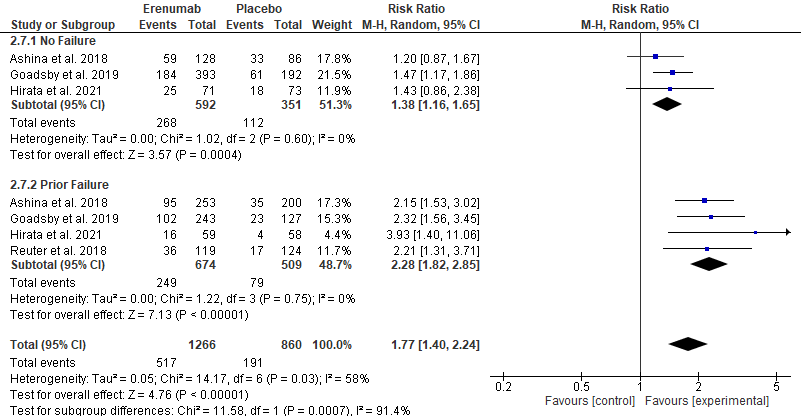


**(C)**

**Figure (S4)** Subgroup analysis based on prior preventive treatment failure status (Prior failure versus No failure). **(A)** MMD at 3 months, **(B)** MSMD at 3 months **(C)** 50% reduction in MMD at 3 months


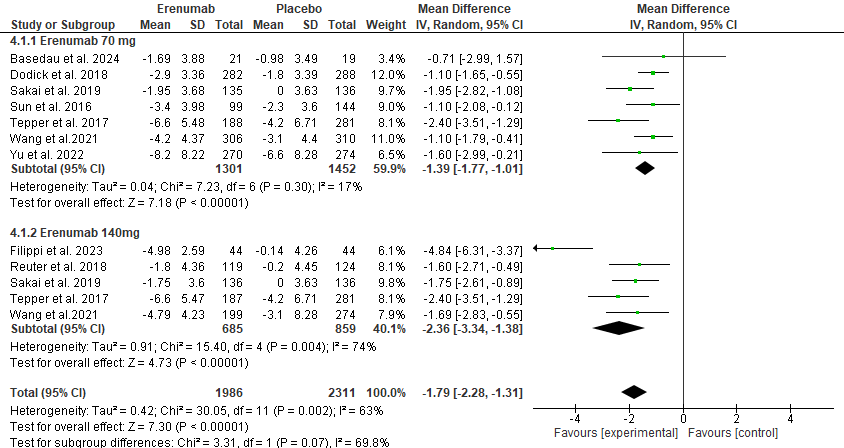


**(A)**


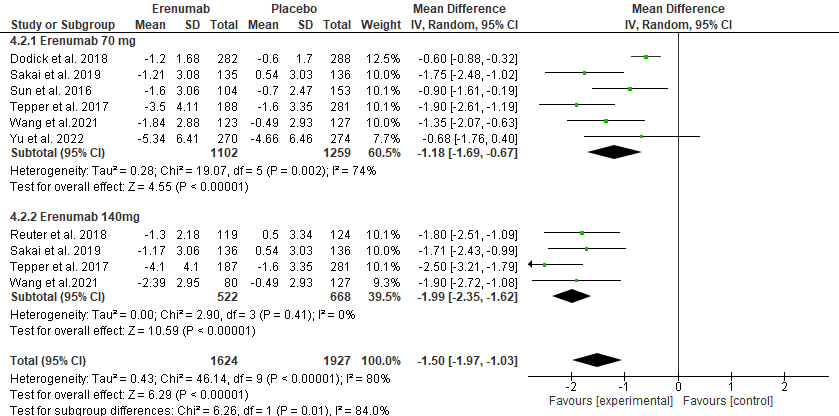


**(B)**


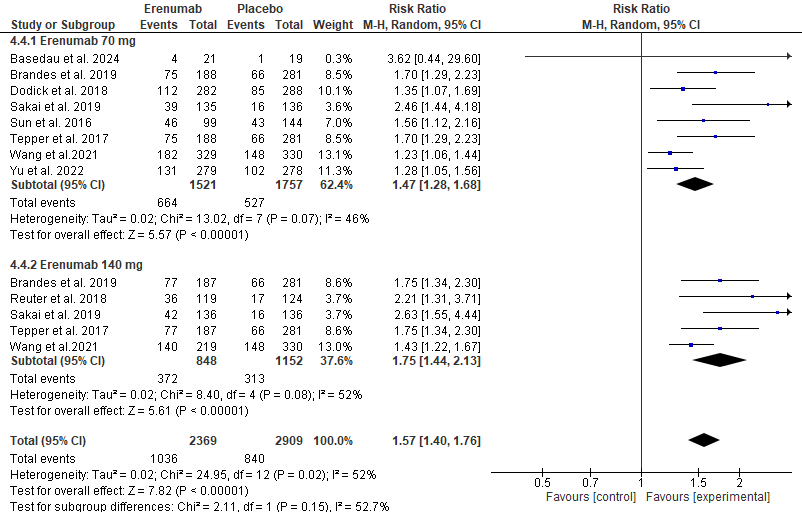


**(C)**

**Figure (S5)** Subgroup analysis based on doses (Erenumab 70mg versus Erenumab 140mg). **(A)** MMD at 3 months, **(B)** MSMD at 3 months, **(C)** 50% reduction in MMD at 3 months.


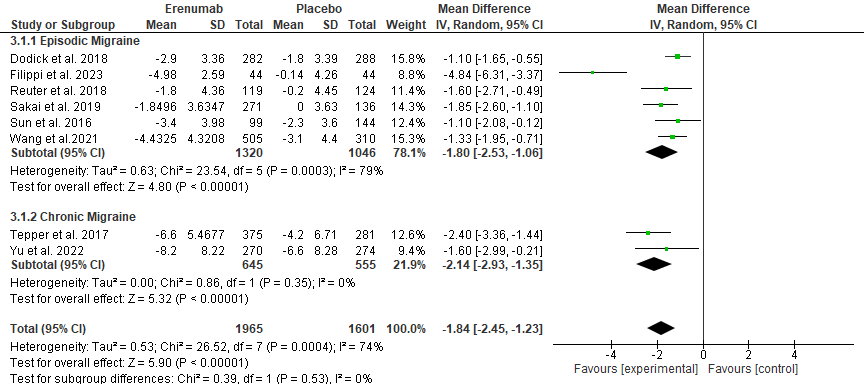

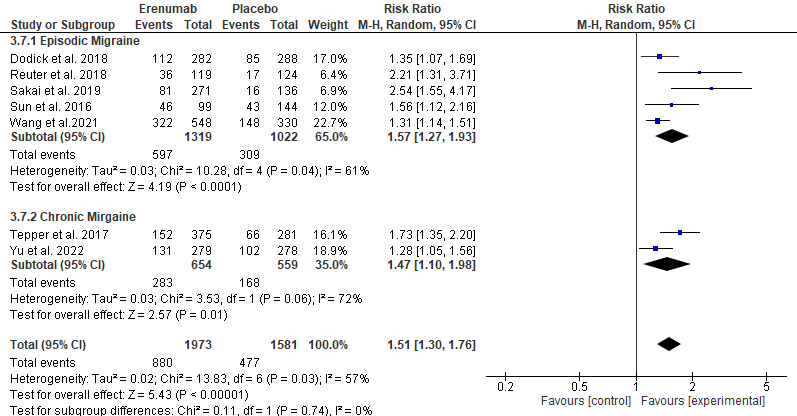


**(A)**

**(B)**

**Figure (S6)** Subgroup analysis based on type of migraine (Episodic versus Chronic). **(A)** MMD at 3 months, **(B)** 50% reduction in MMD at 3 months.


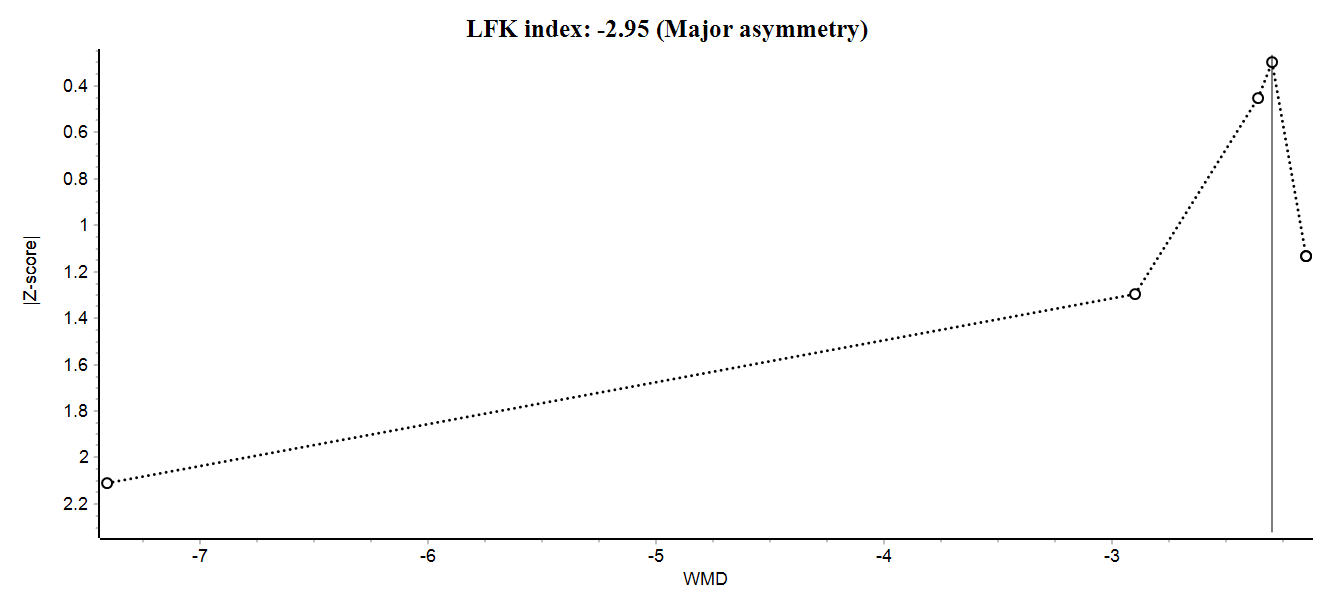

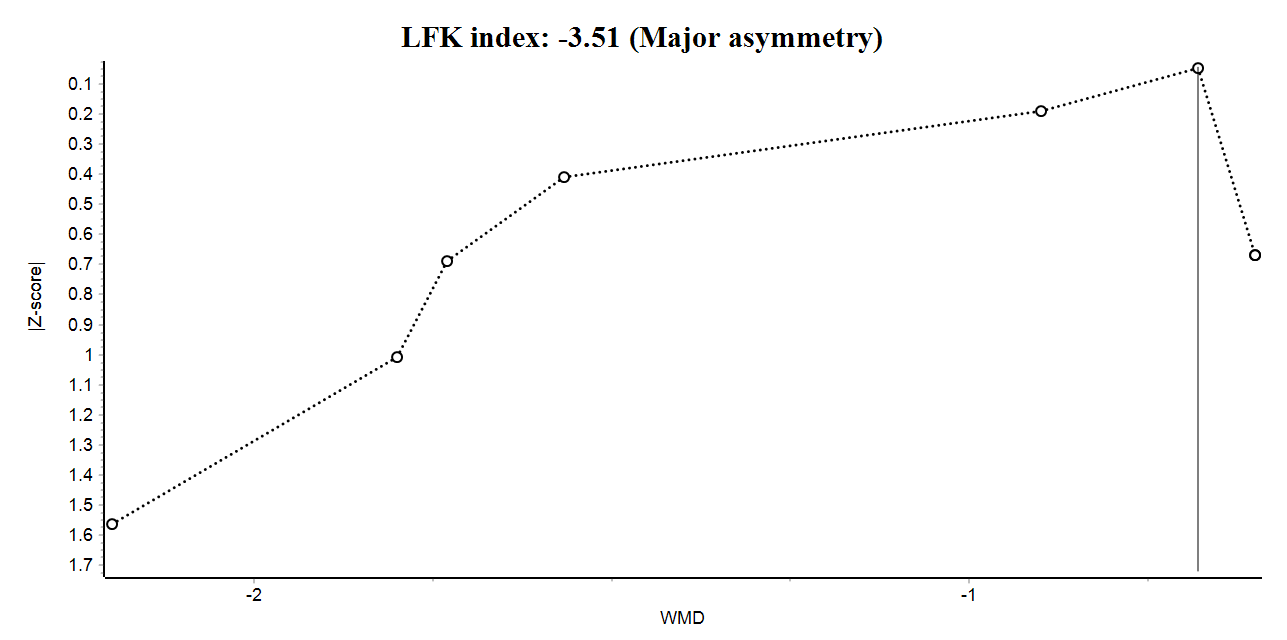

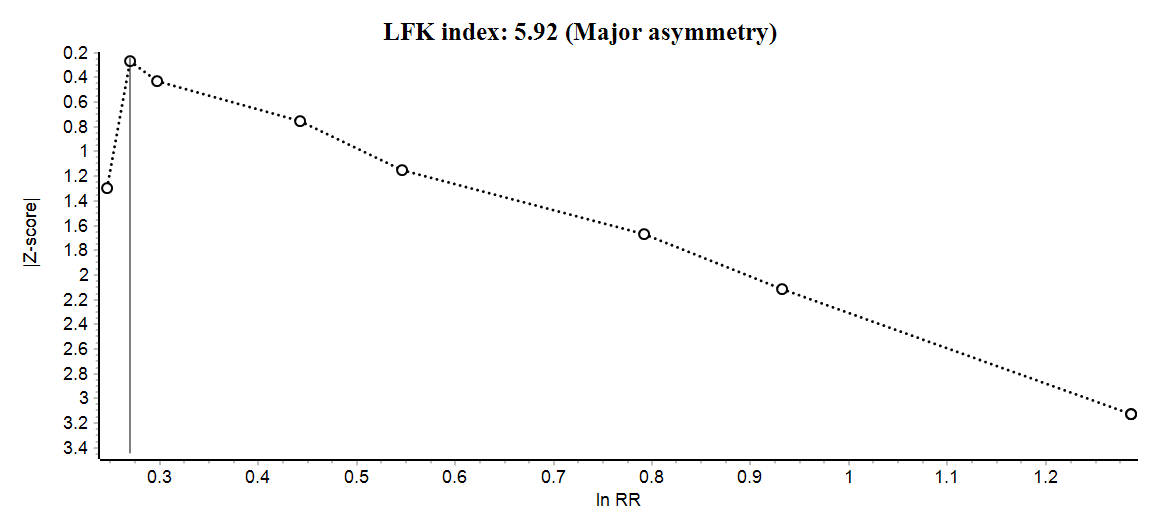


**(A)**

**(B)**

**(C)**

**Figure (S7)** Doi plot and LFK index for **(A)** HIT-6 at 3 months, **(B)** MSMD at 3 months, **(C)** 50% reduction in MMD at 3 months.


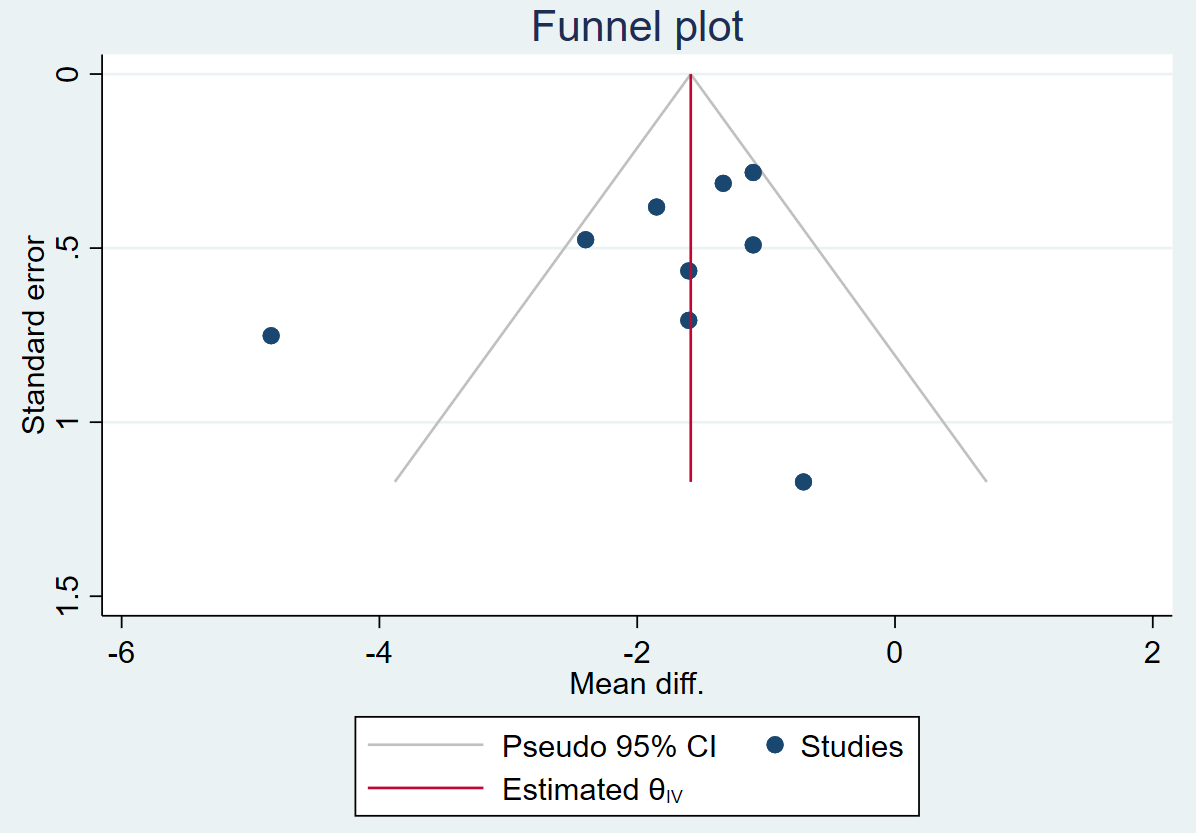


**(A)**


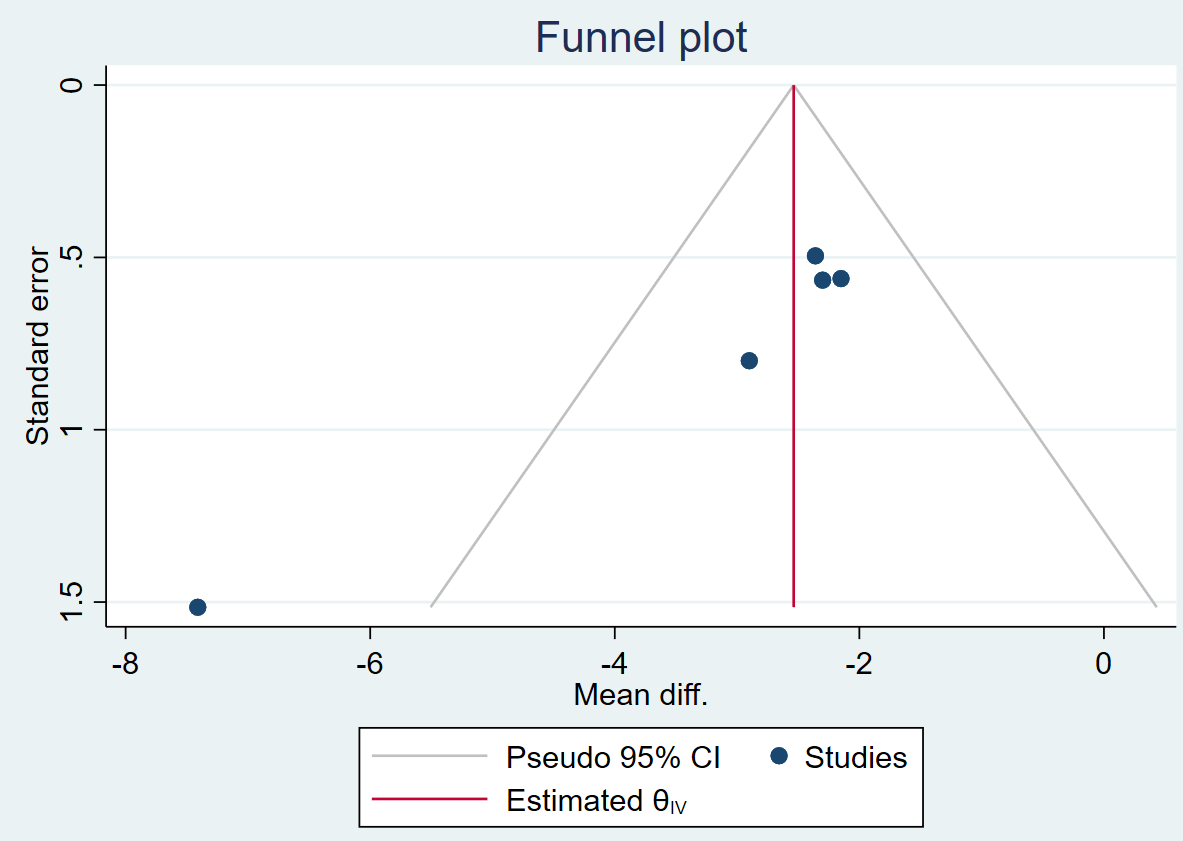


**(B)**

**Figure (S8)** Funnel plots for **(A)** MMD 3 months, **(B)** HIT-6 at 3 months.


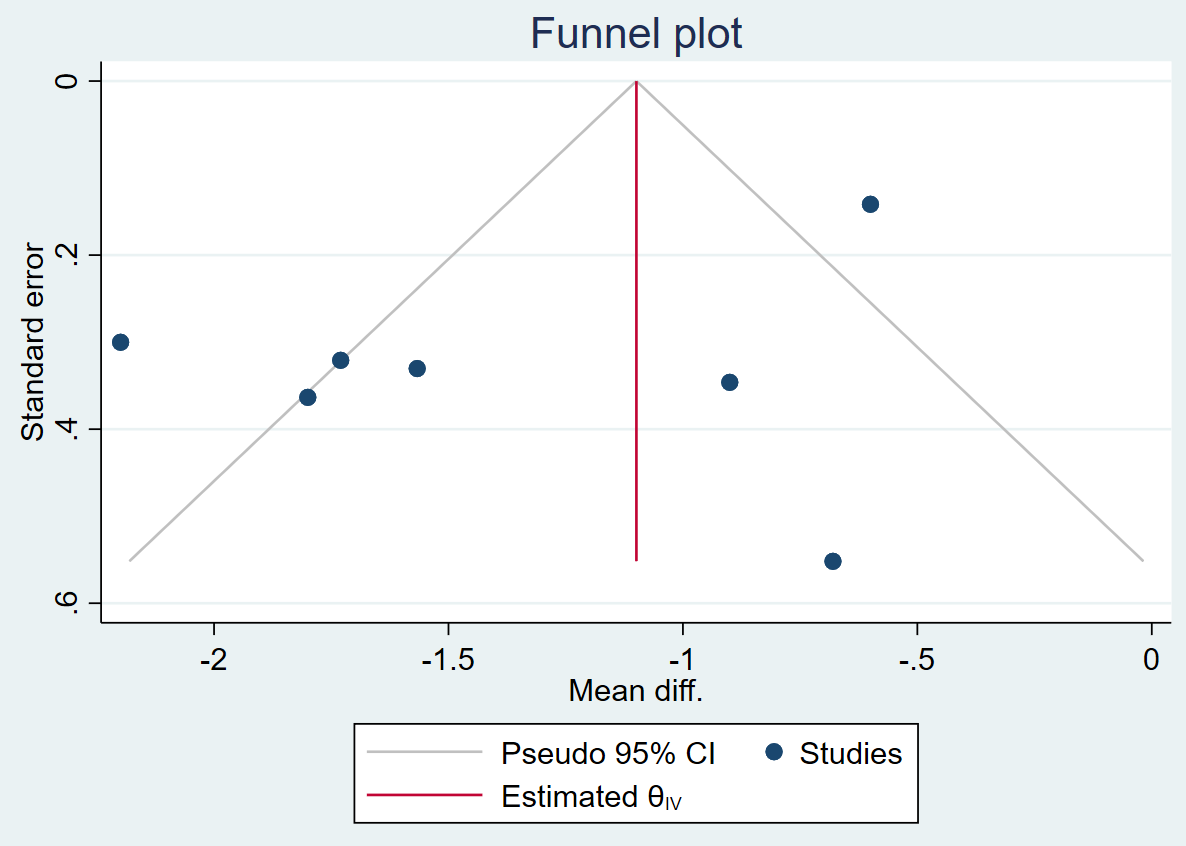


**(A)**


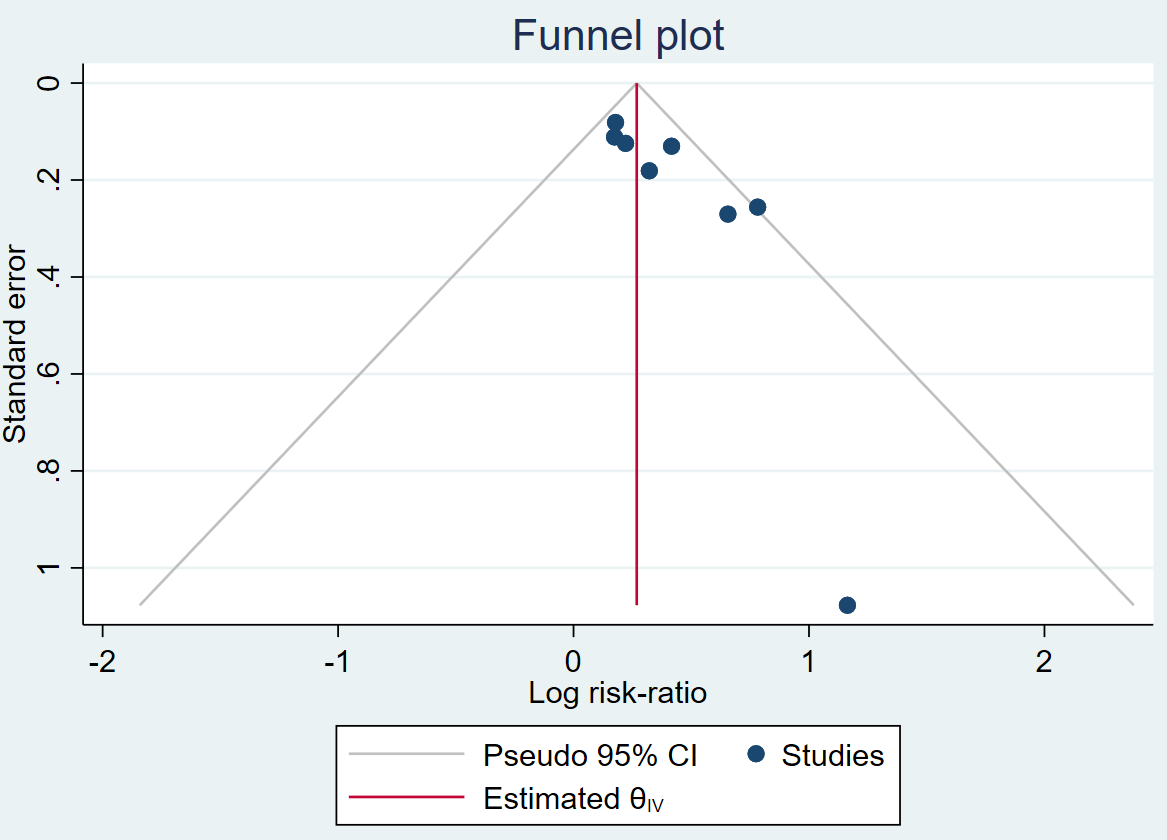


**(B)**

**Figure (S9)** Funnel plots for **(A)** MSMD at 3 months, **(B)** 50% reduction in MMD at 3 months.


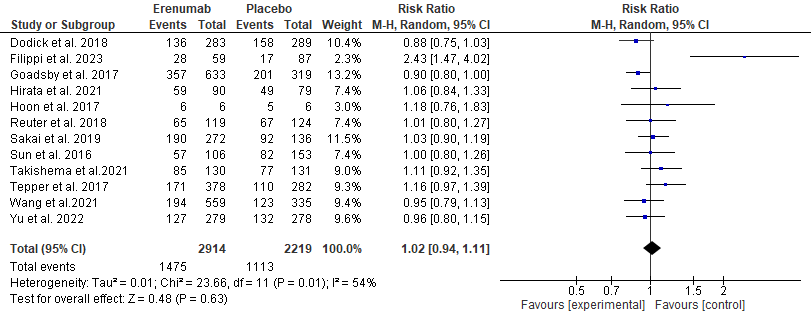


**(A)**

**(B)**

**(C)**


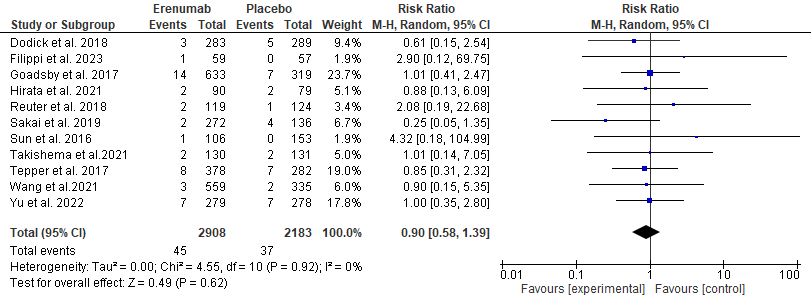

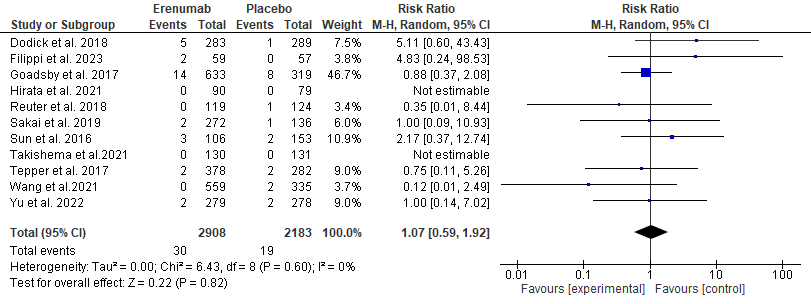


**Figure (S10)** Adverse Events. **(A)** Any adverse event, **(B)** Any serious adverse event **(C)** Any adverse event leading to treatment discontinuation.

**(A)**

**(B)**

**(C)**


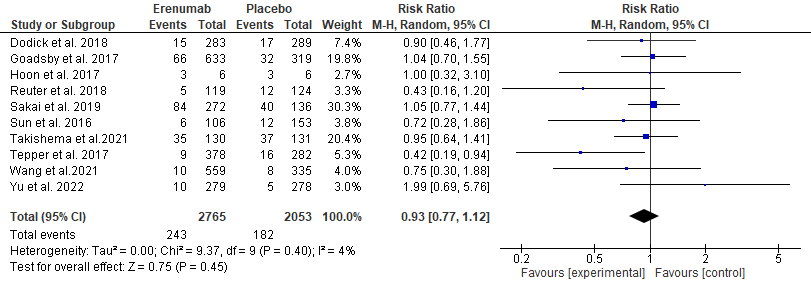

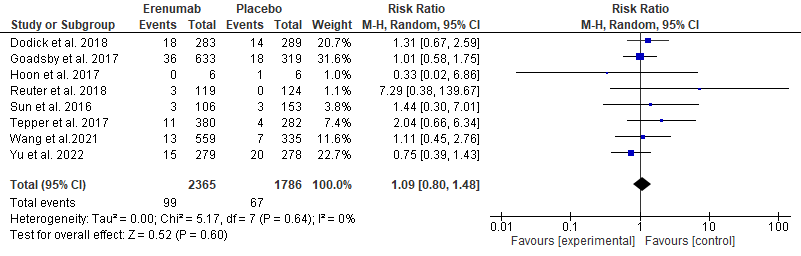

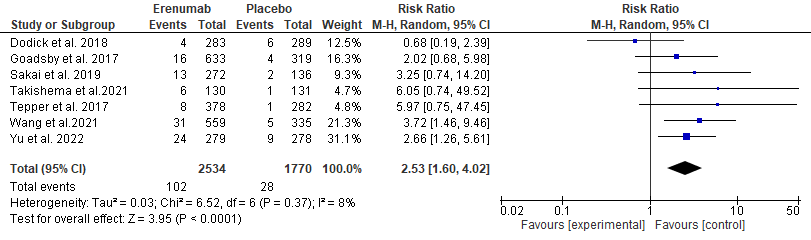


**Figure (S11**) Adverse Events **(A)** Nasopharyngitis **(B)** Upper respiratory tract infection **(C)** Constipation

**(A)**

**(B)**

**(C)**

**(D)**


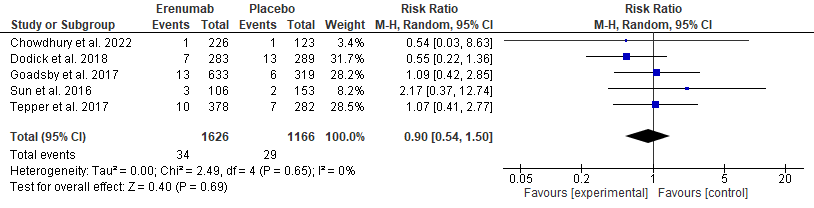

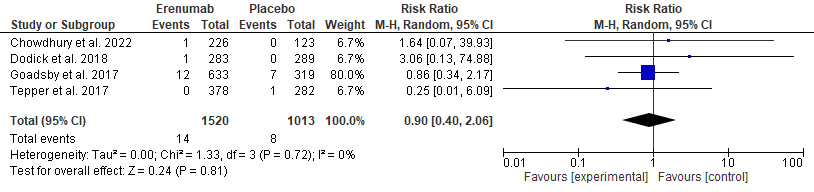

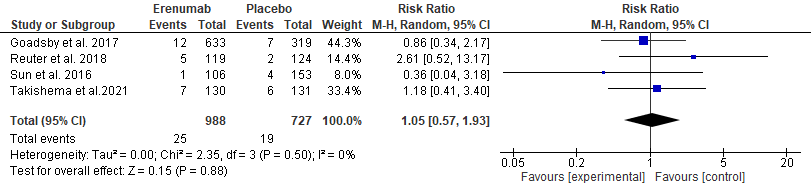

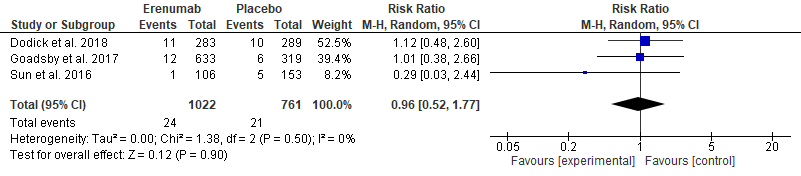


**Figure (S12**) Adverse Events **(A)** Nausea, **(B)** Urinary Tract Infection, **(C)** Back pain, **(D)** Influenza

**(A)**

**(B)**

**(C)**

**(D)**


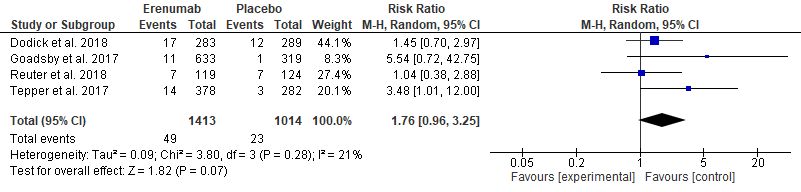

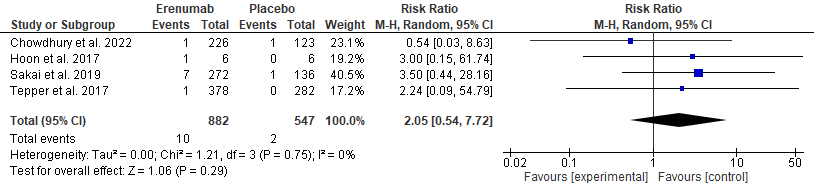

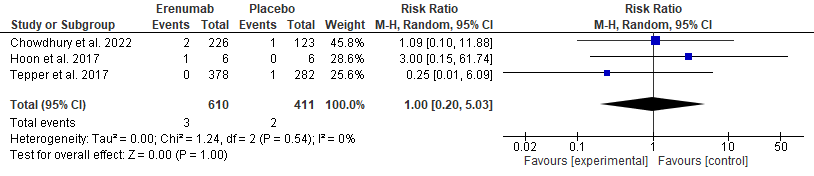

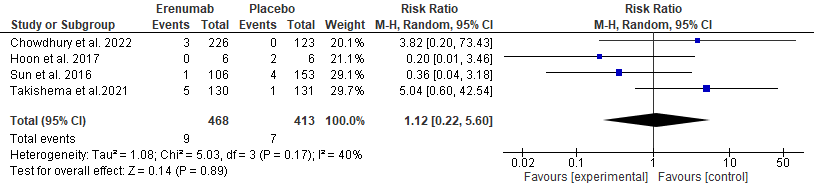


**Figure (S13**) Adverse Events **(A)** Injection site pain, **(B)** Abdominal pain, **(C)** Vomiting, **(D)** Diarrhea.

**(A)**

**(B)**

**(C)**

**(D)**


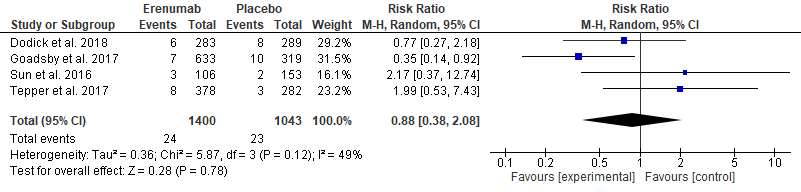

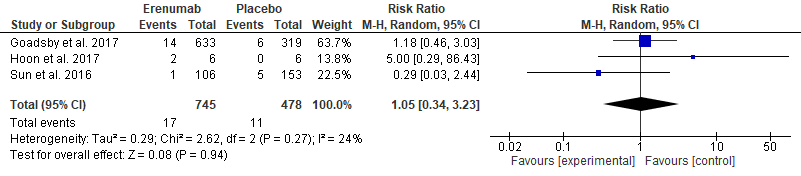

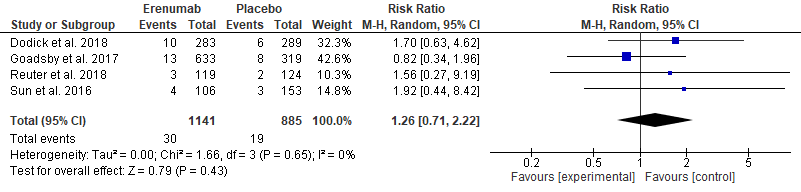

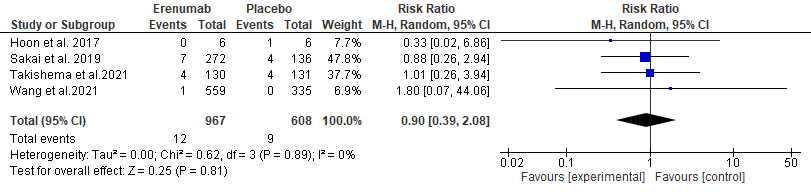


**Figure (S14**) Adverse Events **(A)** Migraine, **(B)** Arthralgia, **(C)** Fatigue, **(D)** Gastroenteritis.
